# Understanding community and patient engagement and involvement (CEI) interventions in acquired brain and spinal injuries (ABSI): a realist review

**DOI:** 10.1136/bmjopen-2025-112463

**Published:** 2026-07-03

**Authors:** Abdel-Rahman Abdel-Fattah, Daniel Sescu, Shobhana Nagraj, Kathryn Jack, Abenezer Tirsit, Madhu N R Kottakki, Almas F Khattak, Angelos G Kolias, Peter J Hutchinson, Charlotte J Whiffin

**Affiliations:** 1 Academic Neurosurgery, University of Cambridge School of Clinical Medicine, Cambridge, UK; 2 NIHR Global Health Research Group on Neurotrauma, Cambridge, UK; 3 School of Medicine, University of Aberdeen, Aberdeen, UK; 4 Department of Public Health and Primary Care, University of Cambridge, Cambridge, UK; 5 East London NHS Foundation Trust, London, UK; 6 Nottingham University Hospitals NHS Trust, Nottingham, UK; 7 Centre for Evidence-Based Healthcare, University of Nottingham, Nottingham, UK; 8 Neurosurgery Division, Addis Ababa University, Addis Ababa, Ethiopia; 9 King George Hospital, Visakhapatnam, India; 10 Department of Community Medicine, Northwest School of Medicine, Peshawar, Pakistan; 11 College of Health and Humanities, University of Derby, Derby, UK

**Keywords:** Health policy, Public health, NEUROSURGERY, Community-Based Participatory Research, Brain Injuries

## Abstract

**Objectives:**

There is growing appreciation of the role of community engagement and involvement (CEI) in designing context-specific interventions for individuals with acquired brain and spinal injuries (ABSI). This realist review evaluates CEI strategies in ABSI research in both high-income (HICs) and low- and middle-income countries (LMICs).

**Design:**

A realist review.

**Data sources:**

Following RAMESES guidelines, a six-stage systematic search of the following databases: Medline, EMBASE, PsycINFO and Global Index Medicus was conducted from inception to March 2025.

**Eligibility:**

Studies assessing CEI in ABSI research design and implementation were included. Neurodegenerative pathologies and studies focusing solely on perspectives or attitudes were excluded.

**Data extraction and synthesis:**

Data were extracted and synthesised using context-mechanism-outcome configurations to explain how CEI strategies were used to improve outcomes.

**Results:**

22 studies were included. Data showed three context-specific focus areas in HICs and LMICs. In HICs prominent contexts were (a) low self-efficacy, (b) untailored rehabilitation services and (c) poor digital literacy. These were addressed through (a) recognising volitional barriers, (b) creating modular programmes and (c) end-user-tested tele-education tools. Whereas in LMICs, the data showed high rates of (d) stigma, (e) poor workforce capacity for community care and (f) barriers to accessible community support. These were addressed through (d) involving community champions, (e) task-shifting addressing workforce gaps and (f) state-level policy changes. Most common CEI interventions in both HICs and LMICs were community advisory boards, Delphi method and integrated knowledge-translation approaches.

**Conclusions:**

This study describes how different contextual factors affecting ABSI populations in HICs and LMICs interact with CEI strategies to trigger mechanisms that improve research engagement and patient outcomes. The study also highlights the dearth of CEI reported in neurosurgical trials. CEI should be embedded in experimental research to account for varying economic, social and infrastructural contexts, particularly in rural and LMIC settings.

**STRENGTHS AND LIMITATIONS OF THIS STUDY**

- This realist review followed the Realist And Meta-narrative Evidence Syntheses: Evolving Standards II guidance and a six-stage iterative approach, to enable thorough assessment of which community engagement and involvement (CEI) interventions work for whom, where and how across different contexts.
- Following the initial programme theory, the search strategy was iteratively expanded to improve capture of studies from low- and middle-income country (LMIC) settings.
- The data were further analysed separately for high-income countries and LMICs, contributing valuable insights to the differences in CEI strategies in different contexts.
- The context, mechanism and outcome configuration framework enabled synthesis of heterogeneous qualitative and mixed-methods evidence beyond traditional effectiveness-focused systematic reviews.
- Interpretation of realist syntheses is limited by inconsistencies in CEI definition and the available published literature which may have influenced study identification and geographical representation.

## Introduction

 Community engagement and involvement (CEI) in neurosurgical research is integral to developing contextually appropriate and population-specific interventions for the care of individuals and communities affected by acquired brain and spinal injuries (ABSI).^1^ Traditional investigator-driven research has often considered patients and communities as passive recipients of care. This approach fails to recognise the health disparities affecting certain populations as well as their priorities, particularly those in rural settings or low and middle-income countries (LMICs).^1^ Therefore, CEI has been defined as an interdisciplinary collaboration between groups of people who share geographical proximity and healthcare interests or issues, to address the physical, social and psychological challenges unique to these populations, in an equitable manner.^2^

Although there has been a growth in global neurosurgical research, there remains a dearth of literature recognising the importance of diverse stakeholder involvement in ABSI research, particularly in LMICs.^3^ This approach is of particular relevance to patients affected by ABSI due to the resultant long-term disability and impact on the physical, psychological and social well-being of the patients, their families and wider community.^4^ Despite advancements in medical technologies and therapeutic approaches for patients with ABSI in the acute phase of their illness, access to neurorehabilitative care remains a significant challenge in rural populations and those in LMICs.^5^ The WHO estimates that only 5%–15% of individuals living with a physical disability in LMICs have access to assistive devices.^5^ A 2018 systematic review of 77 studies illustrated three priority areas affecting access to neurorehabilitation in LMICs: lack of or high cost of transport in urban areas to these facilities; poor health literacy and limited healthcare workforce (eg, <1 rehab professional per 100 000 population in India).^6^

CEI interventions are often grounded in behavioural theories such as self-determination theory (SDT) and social cognitive theory (SCT).^7^ These theories are relevant for people living with ABSI as they often face loss of autonomy, reduced self-efficacy and community marginalisation. A structured CEI approach involving patients and communities in the design and implementation of these interventions may ensure that the interventions are not only effective and appropriate but also aligned with the local priorities. In high-income countries (HICs), the growing use of telehealth technology may introduce barriers to healthcare access, particularly for the older demographic and those living in socioeconomically deprived areas.^8^ ^9^ By involving patients and stakeholders in the design of these technologies, CEI is likely to result in these interventions being user-friendly, accessible and sustainable.^9 10^

To date, there are no realist syntheses examining CEI strategies in the ABSI population, nor the differences across HIC and LMIC settings. The primary aim of this review was to synthesise evidence from ABSI populations in both HIC and LMIC settings, to improve the understanding of CEI interventions currently implemented and highlight priorities for improvement. Compared with a systematic review, a realist review is more suited to handling qualitative literature to convey the mechanisms (M) by which the intervention works, outcomes (O) and contextual factors (C) that lead to it being effective in one context/geographical region and not the other (eg, Chile vs UK, or refractory epilepsy populations vs spinal cord injury patients). In realist literature, these data are often synthesised into context, mechanism and outcome configurations (CMOCs) to better illustrate the relationship between the three factors. As highlighted by the UK Medical Research Council’s guidance on the development and evaluation of complex interventions, a realist approach is an appropriate theoretical approach for understanding which CEI interventions work for whom, where and how.^11^,^13^ It is also one of the complex review types recognised by the National Institute for Health and Care Research (NIHR) Complex Reviews Unit.^13^ The Cochrane’s summary of qualitative evidence synthesis describes the role of realist reviews as fitting in the absence of an explicit programme theory or literature supporting how the implementation of a specific intervention relates to its outcomes.^14^ This is where a realist review or meta-interpretive review can be undertaken.^14 15^

## Methods

This realist review was conducted in line with the six systematic iterative stages of the Realist And Meta-narrative Evidence Syntheses: Evolving Standards (RAMESES) guidelines (online supplemental file 1).^16^ This method helps inform which CEI approaches are to be used when designing future research and interventions for ABSI. The protocol was published with PROSPERO (ID: CRD420250648600).

### Step 1: Clarifying the scope of the review

In the initial stage, the objectives of the review were established:

- To understand how mechanisms triggered by CEI strategies achieved a positive outcome in an ABSI population.
- To identify the contextual factors, in both HIC and LMICs, that influenced the success of these interventions.

Following a scoping search of the CEI literature in ABSI populations, an initial programme theory (IPT) was developed identifying potential causal pathways, mechanisms (including empowerment and knowledge exchange) and contextual factors (including stigma, cultural practices, healthcare infrastructure).

### Step 2: Search for evidence

A comprehensive and iterative three-step search strategy was employed to identify relevant literature in the following databases: Ovid Medline, EMBASE and APA PsycINFO from inception to 18 March 2025. An additional search of Global Index Medicus (GIM) was made, from inception to 28 March 2025, following recognition of the dearth of LMIC literature reported in the former three databases. A manual search of reference lists of relevant systematic reviews was conducted. Grey literature sources were also searched between 31 March and 28 April 2025. These included Google Scholar, policy reports and documentation of international health organisations, for example, WHO, UNICEF, as well as disability and rehabilitation organisations, for example, AO Spine, International Brain Injury Association and WHO Global Rehabilitation Alliance.^17^,^20^ The title, abstract and full-text screening were conducted by two independent reviewers (A-RA-F and DS), and discrepancies addressed by a senior panel (CJW, SN and KJ). The search strategy was structured around two key concepts derived from the research question. The first concept block captures the spectrum of ABSI conditions of interest, and the second focuses on community engagement and participatory research approaches. The full database-specific and grey literature search strategies are available in online supplemental file 2. The inclusion and exclusion criteria are shown in table 1.

**Table 1:** Eligibility criteria

	Inclusion	Exclusion
Population (P)	Patients of all ages with acquired brain and spinal injuries including: Traumatic brain injury: EDH, cSDH, aSDH, SAH Spinal cord injury Intracerebral haemorrhage Acute hydrocephalus Brain or spinal infections Epilepsy	Parkinson’s and dementia
Exposure (E)	Studies assessing CEI in research for a population with ABSI.	Studies assessing patient and community perspectives and attitudes with no CEI in the design or implementation of the research Studies assessing community engagement/societal participation as a clinical outcome
Setting (S)	No limitation on setting/geography No limitation on study types No limitation on language	

ABSI, acquired brain and spinal injuries; aSDH, acute subdural haematoma; CEI, community engagement and involvement; cSDH, chronic subdural haematoma; EDH, extradural haematoma; SAH, subarachnoid haemorrhage.

### Step 3: Appraising and extracting the data

Studies were assessed on their ability to contribute meaningful insights to the IPT, rather than solely methodological criteria. The emphasis was placed on the relevance of the study in providing valuable understanding of the CMOCs of individual CEI strategies. Data were extracted using a predefined extraction framework constructed around CMOCs (online supplemental table 1).

### Step 4: Synthesising evidence and refining programme theories

The evidence was synthesised iteratively to develop and refine CMOCs. The data were assessed with a view to examine how specific contextual factors (eg, public transport infrastructure, cognitive biases/cultural norms) interact with various mechanisms (eg, knowledge exchange, empowerment) to generate a particular outcome (eg, improved patient autonomy or destigmatisation). The theories driving these mechanisms were also examined (tables2  3).

**Table 2:** Summary of CMOCs in HICs

Title of CMOC	CMO configuration with CEI strategy	Underpinning theory with supporting reference
CMOC 1		
Recognising volitional barriers	When physical and psychological barriers (such as lack of confidence or fear) to engaging with self-management of their health are recognised (context), implementing CAB-facilitated rehabilitation planning using HAPA-informed motivational interviewing and action planning (mechanism) can improve autonomy and self-efficacy in managing their own care (outcome).	HAPA,^39 49 50^ SDT^37^
Improving motivation and self-regulation	When patients are encouraged to develop skills for self-regulation and techniques to address their avolition (context), HAPA-informed goal-setting delivered CAB and CBPR-approaches (mechanism) enables patients to remain engaged with the research intervention through an iterative feedback loop (outcome).	HAPA^39 49 50^
CMOC 2		
Addressing patient fear of failure	When failure is framed as a series of obstacles which patients have the tools to address (context), CAB-supported peer-mentorship and co-design of rehabilitation programmes leverage SDT to enable positive reinforcement and peer-encouragement (mechanism). This improves clinical outcomes and self-efficacy (outcome).	SDT^29^
CMOC 3		
Enhancing patient skills in self-advocacy	When patients are empowered to communicate their needs and connect with healthcare teams (context), greater uptake of clinical interventions will occur (outcome) as CoPs facilitate knowledge exchange through IKT approaches enabling patients to develop self-advocacy skills (mechanism).	SCT,^37 38 46^ CoP^39 55 56^
CMOC 4		
Leveraging shared experiences	In an environment where rehabilitation services focus on patient-centred solutions (context), patient care in the local community will be improved (outcome) as knowledge exchange generated through CABs and CBPR approaches has informed the healthcare intervention (mechanism).	CAB,^37 43 61^ participatory co-design,^37 38 46^ SDT,^29 37^ SCT^37 38 46^
Adapting generic tools	When clinical tools are adapted to reflect the specific functional needs of patients (context) through participatory co-design workshops involving patients and communities (mechanism), the tools align better with their lived experiences as they are not only efficacious but contribute to effective and equitable interventions (outcome).	Participatory co-design, health belief model^59^
CMOC 5		
Creating modular rehabilitation programmes	When programmes are constructed in such a way to accommodate varying levels of ability and with differential access to local resources (context), participatory co-design of modular rehabilitation programmes enables HBM-driven flexible and personalised goal-setting (mechanism) leading to improved patient outcomes and engagement (outcome).	HBM, goal-setting theory^37 38^
Embedding peer-mentorship	When CBPR-informed peer-mentorship programmes are incorporated in service design (context), mentors are able to provide tailored emotional and practical support through SCT-described social modelling (mechanism) leading to improved confidence and sustained community participation (outcome).	SCT^37 38 46^
CMOC 6		
Enhancing digital literacy	When TBI and SCI patients face challenges in using telemedicine due to cognitive or physical barriers (context), CAB-led usability testing of digital platforms with Diffusion of Innovation theory-driven iterative feedback loops (mechanism) the platform will be more user-friendly and accessible, which will result in more consistent use and higher satisfaction (outcome).	Diffusion of innovation theory,^62^ participatory co-design, usability theory^37 61^
CMOC 7		
Provision of ongoing support services	When patients face technical challenges on telemedicine platforms (context), CAB-informed telehealth services providing patient-led digital literacy training reduces frustration and builds familiarity and trust as described by normalisation process and cognitive load theories (mechanism), resulting in improving engagement with telemedicine tools (outcome)	Normalisation process theory,^32 37^ cognitive load theory^32^

CAB, community advisory board; CBPR, community-based participatory research; CEI, community engagement and involvement; CMOCs, context-mechanism-outcome configurations; CoP, community of practice; HAPA, health action process approach; HBM, health belief model; HICs, high-income countries; IKT, integrated knowledge translation; SCI, spinal cord injury; SCT, social cognitive theory; SDT, social determination theory; TBI, traumatic brain injury.

**Table 3:** Summary of CMOCs in LMICs

Addressing context	CMO configuration with CEI strategy	Underpinning theory with supporting reference
CMOC 8		
Increasing confidence	When individuals with ABSI face stigma and marginalisation (context), community-champion taskforces and NGO-led public awareness campaigns involving role models and trusted advocates promote a positive narrative around a specific ABSI leveraging SCT-informed social modelling (mechanism) which encourages patients to engage with healthcare services and promotes community reintegration (outcome)	SCT^38 46^
CMOC 9		
Combating misinformation	When communities have misconceptions about neurological conditions such as epilepsy (context), NGOs and community leaders achieve a sense of trust and acceptance through the dissemination of culturally-appropriate health information. This develops SCT-informed normalisation and social learning (mechanism) reducing stigma and enables acceptance of the healthcare intervention.	SCT^38 46^
Promoting role models of behaviour change	When role models with a specific ABSI share a positive outlook and demonstrate healthy behaviours through public events and educational campaigns (context), improved social inclusion of patients occurs (outcome) through community champion-led advocacy campaigns which reinforce positive attitudes through SCT-informed behavioural modelling and observational learning (mechanism).	SCT^38 46^
CMOC 10		
Training rural healthcare workers	When LMIC healthcare systems face rural healthcare workforce shortage (context), the co-design of local training programmes supports culturally-tailored skillset development through BLT-informed mentorship and collaborative learning methods (mechanism) improves outcomes in underserved areas (outcome).	BLT^28^
Addressing workforce shortages through task shifting	When healthcare workforce shortages limit access to ABSI care in LMIC settings (context), Delphi-based consensus development facilitates task-shifting models driven by collective decision making as described by theory of planned behaviour (mechanism). This expands the service and bridges unmet need (outcome).	Task-shifting models^29^
CMOC 11		
Incentivisation	When professional recognition and career growth opportunities are limited (context), workforce satisfaction and retention can be increased (outcome) through CEI-informed workforce incentive programmes increasing commitment and SDT-informed autonomy as they feel valued and recognised (mechanism)	Incentive-based workforce models^30^
CMOC 12		
Aligning policy with local need	When the local infrastructure does not support access to care for ABSI patients (context), CoP-driven multistakeholder taskforces can create sustainable healthcare policies through diffusion of innovation-informed knowledge exchange (mechanism). This improves service accessibility (mechanism).	Diffusion of innovation theory,^62^ SDT, CoP, advisory meetings,^82^ community champions^26 28 30^
Breaking down social reintegration barriers	When patients with ABSI face barriers to community re-integration (context), the co-design of rehabilitation programmes through participatory workshops and advisory boards helps identify and prioritise community-specific barriers through SDT-informed ownership and autonomy (mechanism) resulting in improved community reintegrative support (mechanism)	Participatory co-design^37 38 46^
Expanding rural healthcare reach	When geographical barriers to ABSI care exist (context), IKT-driven initiatives support the development of locally adapted services, eg, mobile clinics (mechanism), (mechanism) ensuring essential services are accessed by remote populations (outcome).	Integrated knowledge translation^27 39^

ABSI, acquired brain and spinal injury; BLT, blended learning theory; CEI, community engagement and involvement; CMOC, context-mechanism-outcome configuration; CoP, community of practice; IKT, integrated knowledge translation; LMICs, low and middle-income countries; NGO, non-governmental organisation; SCT, social cognitive theory; SDT, social determination theory; SET, social exchange theory.

To recognise how CEI strategies differ between HICs and LMICs, emphasis was placed on understanding why and how different strategies were used in different contexts. Included studies were categorised into HIC and LMIC groups according to the World Bank income classification criteria.^21^ As evidence accumulated, emerging insights were used to refine the IPT and recognise the complex, often bidirectional or tridirectional, relationships between the three components of the CMOC.

In this synthesis, the term ‘mechanism’ is used to describe the psychological or social processes triggered by various intervention strategies (eg, CABs or CBPR) to generate specific outcomes.

### Step 5: Developing explanatory models

The refined CMOCs were synthesised into one programme theory, stratified by HIC and LMIC, to generate actionable insights pertaining to CEI interventions helpful in each context. Thereafter, a visual model was developed to illustrate causal pathways and the interplay between context, mechanism and outcomes to harmonise the understanding of these complex relationships.

### Step 6: Validation and dissemination of findings

The CMOCs were iteratively reviewed by a panel of experts in realist reviews (SN and KJ), including those with experience of realist methods (SN and KJ) to develop an overall programme theory. The programme theories were validated through cross-comparison with HIC and LMIC examples from case studies in the literature. Findings from this study will be shared locally, nationally and internationally through webinars, conferences, and professional social media networks (such as LinkedIn).

### Patient and public involvement

In recognition of the importance of patient and public involvement (PPI) in ABSI research, this study was informed by previous PPI case studies and workshops led by authors AFK, CJW, PJH, AGK and SN.^22^ As this was a review, PPI was not included in the design or conduct of the study. Nevertheless, the expertise of ABSI researchers in LMICs was sought throughout (AT, AK and AFK). Dissemination of the results of this study will include NIHR ABSI Grant collaborating groups in both HIC and LMICs.

## Results

### Search results

The search identified 766 articles as shown in online supplemental figure (Preferred Reporting Items for Systematic Reviews and Meta-Analyses (PRISMA) flow chart). Initial scoping searches identified several ‘sibling’ papers (papers providing additional detail on interventions discussed in the literature) which helped support the development of initial programme theories and temporal CMOC trends.^23^,^25^ Of the 766 studies, 75 underwent independent full-text screening. Most studies excluded (n=42) did not reflect explicit methodology of community engagement (rather they were simply assessing community participation in qualitative research). Therefore, following consultation with the expert CEI panel, studies were only included if they reflected an objective degree of engagement with patients and/or communities as defined by step 4 and above in UNICEF’s community engagement model (online supplemental file 3).^25^ A total of 22 studies met the inclusion criteria and were included in the realist synthesis. Initially, 17 studies were included from Ovid Medline, EMBASE, APA PsycINFO databases. Following the addition of the GIM bibliographic database, a further five studies (all of which were from LMICs) were included in the refined dataset for CMOC synthesis based on inclusion criteria as well as the theoretical grounding of the IPT below.^26^,^30^ No literature eligible for inclusion was found from grey literature sources. Full details of search strategy output are available in online supplemental file 2.

### Characteristics of included studies

A summary of characteristics is illustrated in table 4. Studies from HICs (n=17) were conducted in the USA (n=6),^31^,^36^ Canada (n=6),^37^,^42^ Sweden (n=1),^43^ Switzerland (n=1),^44^ Ireland (n=1)^45^ and Australia (n=2).^46 47^ HIC studies primarily addressed SCI (n=14)^31^,^3335^ and traumatic brain injury (TBI) (n=3).^34 46 47^ Four studies focused on technology-driven solutions^32 36 38 44^ and 13 on patient autonomy/self-efficacy.^3133^,^35 37 39^ CEI strategies varied from participatory co-design,^37 38 43 46^ community advisory boards (CABs)^31 32^ and health action process approaches (HAPAs).^39^

**Table 4:** Characteristics of included studies

Study	Year	HIC/LMIC	Findings summary	Theories identified	Disease focus
Studies from HICs					
Carroll *et al*^45^	2024	HIC	CBPR exploring patient involvement in preclinical SCI research to promote inclusion and relevance.	Self-determination theory	SCI
Benn *et al*^38^	2023	HIC	Co-design approach to develop FES/VFBT rehabilitation prototype in Canada.	Self-determination theory; social cognitive theory; empowerment theory	SCI
Newman *et al*^32^	2023	HIC	CAB to develop telehealth/self-education platform for SCI patients in the USA.	Health belief model; self-efficacy theory	SCI
Biller *et al*^33^	2023	HIC	CoP approach integrating SCI community members into research design to promote relevance and trust.	Community of practice; self-determination theory	SCI
Walsh *et al*^31^	2022	HIC	CAB approach to improve medical rehabilitation in SCI patients in the USA.	Self-determination theory; experiential learning theory; social exchange theory; goal-setting theory	SCI
George *et al*^46^	2022	HIC	CBPR to provide mobility support for TBI/SCI in Australia.	Social cognitive theory; self-efficacy theory; behaviour change theory	TBI/SCI
Hitzig *et al*^40^	2021	HIC	Co-design process to develop national community participation indicators for SCI rehabilitation services in Canada.	Knowledge translation theory; self-efficacy theory; participatory action research	SCI
Alavinia *et al*^41^	2021	HIC	Participatory design to create employment indicators supporting SCI patient reintegration into the workforce.	Self-efficacy theory; participatory co-design	SCI
Bateman *et al*^42^	2021	HIC	Collaborative quality improvement model (SCI IEQCC) used to implement evidence-based indicators across sites.	Empowerment theory	SCI
Douglas *et al*^36^	2021	HIC	CBPR-informed robotic gait training intervention co-designed with multidisciplinary stakeholders for inpatient SCI rehabilitation.	Participatory action research; social learning theory; self-determination theory	SCI
Fiordelli *et al*^44^	2020	HIC	Participatory co-design of a self-management app for pressure injury prevention among SCI patients.	Health literacy theory; knowledge translation	SCI
Wolfe *et al*^37^	2019	HIC	Nationwide CoP to establish national policy changes according to needs of SCI patients in Canada.	Self-determination theory; social cognitive theory; theory of planned behaviour	SCI
Ma *et al*^39^	2019	HIC	HAPA and IKT process to improve physical activity in SCI in Canada.	Self-determination theory; health action process approach	SCI
Newman *et al*^32^	2014	HIC	CBPR-based peer navigator intervention to reduce secondary conditions and enhance community participation.	Social cognitive theory	SCI
Lindberg *et al*^43^	2013	HIC	Co-design to develop PPRQ rehabilitation questionnaire for SCI patients in Sweden.	Self-determination theory; social cognitive theory	SCI
Gauld *et al*^47^	2010	HIC	Action research to develop culturally relevant services for Aboriginal Australians with ABI.	Empowerment theory; social learning theory	ABI
De Pompei *et al*^34^	2001	HIC	Interagency collaborative planning group to coordinate care for people with TBI in Ohio.	Network governance theory; social exchange theory	TBI
Studies from LMICs					
Segovia *et al*^30^	2020	LMIC	LICHE NGO to help destigmatise epilepsy in Chile.	Social cognitive theory	Epilepsy
Tatli *et al*^28^	2019	LMIC	Co-design approach to adapt the WHO ICF tool for SCI patients in Turkey.	Self-determination theory	SCI
Feniman *et al*^29^	2017	LMIC	Delphi approach to develop adapted QoL questionnaire for patients with SCI in Brazil.	Theory of planned behaviour	SCI
Tambourgi *et al*^27^	2013	LMIC	Collaborative framework to improve legislation for patients with epilepsy in Brazil.	Diffusion of innovation theory	Epilepsy
Min *et al*^26^	2003	LMIC	Multistakeholder taskforce to address stigma and care needs in epilepsy patients in Brazil.	Social cognitive theory	Epilepsy

ABI, acquired brain injury; CAB, community advisory board; CBPR, community-based participatory research; CoP, communities of practice; FES/VFBT, functional electrical stimulation plus visual feedback balance training; HAPA, health action process approach; HIC, high-income country; ICF, International Classification of Functioning, Disability and Health; IEQCC, The Spinal Cord Injury Implementation and Evaluation Quality Care Consortium; IKT, integrated knowledge translation; LICHE NGO, The Chilean League against Epilepsy non-governmental organisation; LMIC, low- and middle income country; PPRQ, Patient participation in Rehabilitation Questionnaire; QoL, quality of life; RGT, robotic gait training; SCI, spinal cord injury; TBI, traumatic brain injury.

Studies from LMICs were conducted in Brazil (n=3),^26 27 29^ Chile (n=1)^30^ and Turkey (n=1).^28^ LMIC studies primarily addressed epilepsy^26 27 30^ and complications of SCI in the community.^28 29^ Two studies focused on systemic barriers,^26 30^ one on healthcare access^27^ and two on workforce limitations.^28 29^ CEI strategies in LMICs included formal community champion taskforces,^26 28 30^ family representative groups^27^ and Delphi approaches.^29^

### Initial programme theories

**Figure 1:**
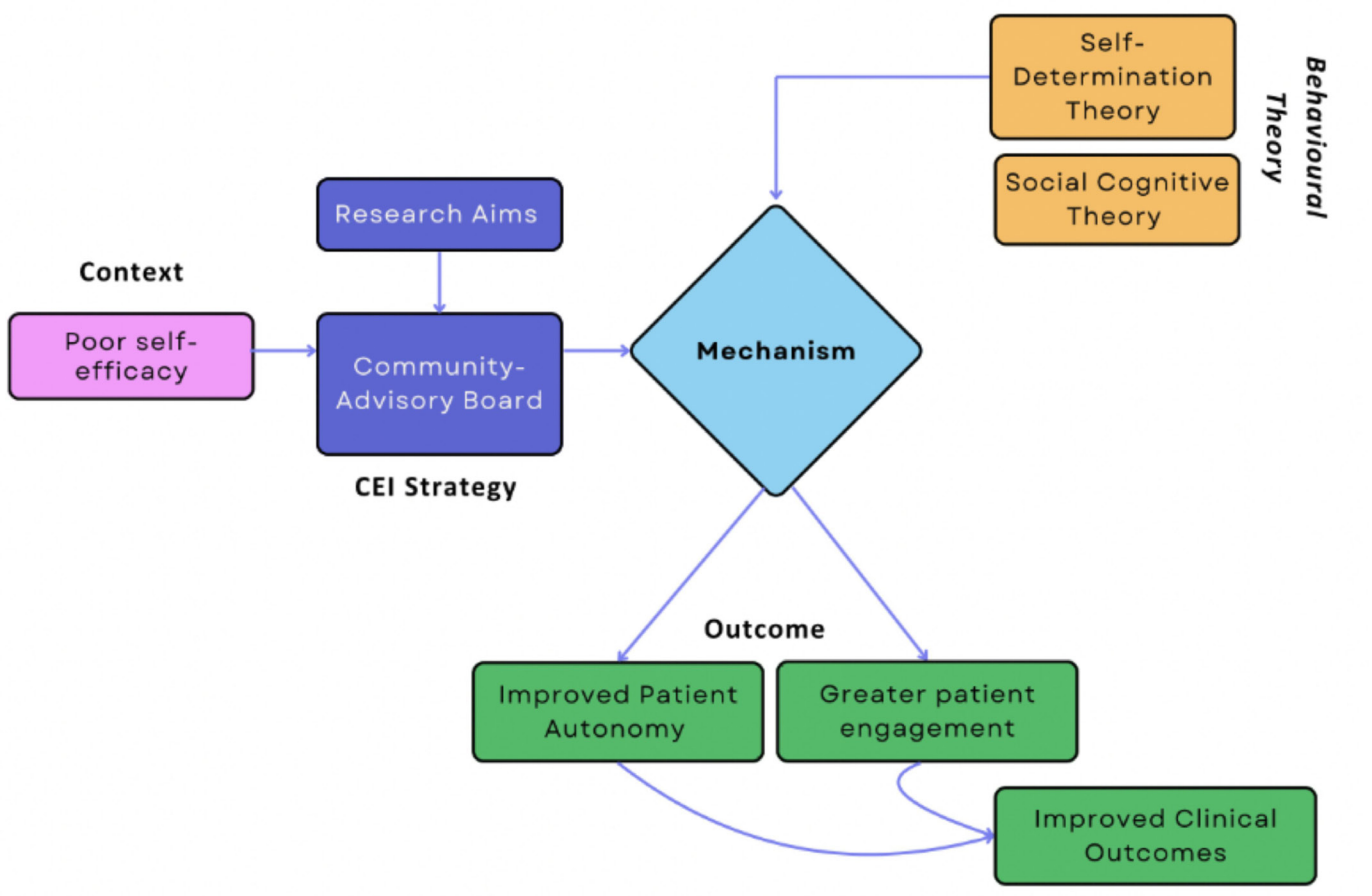
Initial Programme Theory based on example by Walsh *et al*. CEI, community engagement and involvement.

An overall IPT was developed following initial scoping search by identifying potential causal pathways, mechanisms (eg, empowerment or knowledge exchange) and contextual factors (eg, sociocultural practice or healthcare infrastructure). Further iterative searches were then conducted, and articles assessed against the inclusion criteria and/or their relatedness to the IPT (see figure 1). A pattern was extrapolated from the initial articles assessed, yielding the assumption that participatory-research approaches allowed for tailored health interventions that align with specific sociocultural and healthcare contexts.^31 46^ Initial themes drawn included trust-building, iterative feedback and diverse stakeholder involvement to ensure sustainability and impact. The IPT was augmented by several theories applied in the initial studies. For example, Walsh *et al* recognised how the SDT played a role in rehabilitation adherence in SCI patients because their involvement in a CAB improved their intrinsic motivation.^31^ The theories had not yet been stratified by HIC and LMIC due to the paucity of LMIC literature encountered prior to the searching of the GIM database.^26^,^30^

For each study, CMOCs explored how different CEI strategies were implemented in various settings (online supplemental table 1). They also summarise the corresponding theories, some of which are rooted in the behavioural psychology evidence-base, which underpin their effectiveness.

### Programme theories in HICs

#### Refined CMOCs

The broad CMOCs initially identified in each study were analysed and refined based on shared mechanisms into twelve refined CMOCs. These were further stratified by those most identified in literature from HICs (table 2) and those mostly found in the LMIC studies (table 3). Seven CMOCs were identified from HIC studies with the overarching contexts of: (a) addressing low self-efficacy, (b) improving rehabilitation services and (c) training patients to use telemedicine. Five CMOCs were identified from LMIC studies with the overarching contexts of (a) reducing stigma, (b) improving workforce capacity and (c) improving access to care. In realist terms, CMOCs may alternatively be expressed as causal relationships using an ‘If, then, because’ statement. An online supplemental table 2 was synthesised for visualisation of the programme theories.

#### Addressing low self-efficacy

##### CMOC 1: Recognising volitional barriers and improving self-regulation

Individuals with SCI and TBI face physical and psychological barriers to engaging with post-trauma rehabilitation interventions and with community reintegration.^31^ One explanation is a lack of confidence and perceived inability to control their condition which can lead to reduced engagement in self-management therapies and consequently dependence on community healthcare support (context 1 (C1)).^48^ Using CABs to inform research in TBI and SCI rehabilitation planning, studies ensured that patient voices were heard and were central to the co-development of self-management or rehabilitation tools (eg, Functional Electrical Stimulation which reinforced autonomy-supporting interventions mechanism 1).^38^ HAPA is one behavioural theory which several papers have leveraged to achieve this.^39 49 50^ This involved encouraging patients to recognise their own motivation for change (motivational interviewing) and structuring goal-oriented behaviour through iterative patient-engagement.^50^ This relationship built trust between both patients, researchers and healthcare teams and resulted in improved self-efficacy for SCI and TBI patient and caregiver communities (outcome 1 (O1)).

Within the same context, George *et al* used the CBPR approach demonstrating that this CEI strategy helped integrate patient and healthcare professionals into the research team with an emphasis on iterative feedback loops. This ensured patients remained engaged and improved their intrinsic motivation to work on their mobility post-TBI (M1b).^46^ This approach not only bridged the gap between efficacy and effectiveness; it also safeguarded the long-term effectiveness of the intervention through empowering patients to drive iterative change.^51^

##### CMOC 2: Addressing patient fear of failure

Avoidance behaviour secondary to fear of failure and setbacks differentially affects patients with TBI and SCI (C2).^51 52^ Based on current literature, framing failure as a learning process and making stepwise successes increased confidence and reduced fear.^53^ Two studies implemented CABs as their CEI strategy^37 43^ which led to policy changes ensuring healthcare teams offered structured peer-mentorship to navigate setbacks effectively (M2/O2a).^54^ This in turn drove higher self-efficacy as patients developed resilience when seeing peers overcome failure—resulting in a continuous cycle of positive reinforcement (O2b). It also ensured patients remained engaged in therapy despite setbacks (O2c).

##### CMOC 3: Enhancing patient skills in self-advocacy

Individuals with TBI often feel disenfranchised and struggle to communicate their needs (C3).^38^ Through CoPs, research groups with diverse stakeholders were able to translate knowledge through integrated knowledge translation (IKT) approaches into useful and practical advocacy skills.^39^ The CoP model embedded elements of SCT by creating a platform for shared knowledge. It also empowered patients to confidently share their views with clinicians and researchers through peer-led discussions (M3).^37^ Findings from several studies supported the hypothesis that individuals with SCI who learn self-advocacy skills through CoPs developed better engagement with post-injury rehabilitation (O3).^55 56^

### Improving rehabilitation services

#### CMOC 4: Leveraging shared experiences and peer-mentorship

Traditional healthcare approaches often lack peer support, particularly in decentralised healthcare systems (C4a).^57^ George *et al* suggested that patients recovering from TBI/SCI benefitted from peer-led knowledge exchange in addition to clinical expertise.^46^ A collaborative approach was prioritised, using CBPR, with patients regarded as co-researchers fully integrating peer-perspective and experience. Rehabilitation policies were designed in such a way that prioritised patient networking.^37 38 46^Bandura *et al* suggested that social learning (in SCT) included observational learning and peer-mentorship, which when implemented in two of the included studies, fostered a sense of belonging in TBI and SCI patients (M4a).^38 46 58^ One study drew on this CMOC relationship by recognising that creating a sense of relatedness (one of the three components of the SDT) through shared experience promoted self-engagement with rehabilitation services (O4a).^37^

#### CMOC 5: Creating modular rehabilitation programmes and adapting generic tools

Patients with SCI and TBI have differing levels of mobility, pain thresholds and differential access to resources (C5). This requires patient-tailored rehabilitation approaches.^38^ Individuals were found to be more likely to engage in their programme if they perceive it as relevant and adaptable to their needs (health belief model) (M5/O5).^59^ Additionally, two studies leveraged Locke and Latham’s goal-setting theory of ensuring goals are realistic and achievable.^37 38 60^ Participatory co-design of modular rehabilitation programmes enables health-belief model-driven goal-setting which is personalised and flexible. This provided patients a sense of control while limiting long-term attrition.^37 38 43 46^

### Patient training in tele-medicine

#### CMOC 6: Enhancing digital literacy

People living with TBI face unique barriers to engaging with telemedicine modalities due to cognitive deficits in multiple domains, commonly executive function and memory (C6).^32^ Additionally, many interventions have poor uptake due to the lack of patient-centred considerations unique to their target TBI population.^46^ By implementing a participatory co-design or CBPR approach, patients were involved not only in usability testing but were engaged as digital health co-researchers through iterative feedback loops (Diffusion of Innovation theory (DIT)), ensuring that the platforms met both content and user-interface requirements (M6a).^32 37^ Additionally, one study highlighted the role of CABs in empowering expert patients to spearhead patient-led digital literacy workshops which helped end-users understand how to best interact with the platform. This facilitated direct and personable engagement, with patients sharing their experiences (usability theory) (M6b).^37 61^ With complex interventions such as digital technology the evidence supports the use of a stepwise feedback loop combining exposure, education and feedback with patients and researchers to improve digital literacy and sustained engagement (DIT) (O6).^62^

#### CMOC 7: Provision of ongoing support services

Patients with SCI and TBI often lack long-term engagement with digital interfaces commonly due to lack of real-time assistance and technical difficulties (C7).^32^ Several studies demonstrated that the cognitive load associated with overwhelmingly complex interfaces and lack of guidance can be overcome using two methods. First, the co-design of simplified and intuitive interfaces to better understand thresholds of cognitive overload. Second, involving patients and digital-health experts in CABs embedded telemedicine services in all SCI/TBI community care interventions with ongoing technical support. These methods build familiarity and trust as described by normalisation process theories (M7).^32 37^ This improved patient retention led to greater familiarity with the platforms and reduced cognitive load over time, encouraging long-term engagement (O7).

### Programme theories in LMICs

#### Reducing stigma

##### CMOC 8: Increasing confidence and promoting role models of behaviour change

Individuals with ABSI and epilepsy often face social isolation and marginalisation (C8).^26 30^ Several studies support the use of CBPR such as community-champion taskforces such as: The Chilean League against Epilepsy (LICHE) and similar non-profit organisations in clinical research to contribute valuable insights into optimal methods of reintegration into work, education and social life (M8a).^30^ Additionally LICHE NGO-led campaigns with community champions (respected local leaders, teachers, religious figures) have helped normalise engagement and shift public perceptions towards epilepsy and SCI. This pattern of observation, imitation and reinforcement, when driven by trusted figures, was shown to reduce fear and stigma (social cognitive theory) (O8).^26^ Physical settings were also regarded as important factors in enhancing confidence and perceived self-control over health decisions, as positive narratives were more easily reinforced in spaces which felt inclusive and safe to patients (M8b). This was made more achievable by ensuring patient representation in the team delivering the interventions.^26^

##### CMOC 9: Combating misinformation through community education

On average, there is a lower level of health literacy about ABSI among many LMIC populations, often affected by various cultural beliefs (C9).^63^,^66^ One example of this is the belief that disabilities including epilepsy are associated with supernatural causes rather than structural epileptogenic foci.^65^ One study showed that this led to apprehension around medical treatments offered and mistreatment, as complementary remedies including spiritual and traditional healers are seen as the viable option.^27^ Proposed nationwide collaborative frameworks including non-governmental organisation (NGOs), policymakers and community leaders achieve a sense of trust and acceptance through the dissemination of culturally appropriate health information. These also strengthened the influence on government legislation for people living with epilepsy bringing about the widespread delivery of education campaigns to disseminate accurate health knowledge in the Brazilian and Chilean populations. ^29 30^ These studies reported greater acceptance and uptake of modern evidence-based treatments, through SCT-informed social learning and normalisation (M9). Consequently, more individuals trusted the nationwide movements promoting a chain of further healthcare awareness, destigmatisation and health-seeking behaviour in the wider ABSI community, leading to better clinical outcomes (O9).

### Improving workforce capacity

#### CMOC 10: Training rural healthcare workers and task-shifting

Many LMICs are differentially affected by a lack of subspeciality-trained medical professionals in SCI care with much more limited access to community and tertiary neurosurgical care in rural populations compared with that in HICs (C10).^28^ One study co-designed a local training programme to support culturally tailored skillset development through blended-learning theory-informed mentorship and collaborative learning methods (M10a).^28^ This allowed them to provide scalable training to local professionals in unique aspects of care delivery for local ABSI populations.^28^ Tatlı *et al* emphasised the role of a co-design CEI approach, used to adapt a WHO International Classification of Functioning, Disability and Health tool. This is in recognition of the uniquely limited access to SCI inpatient rehabilitation experienced by the majority of the Turkish population (with only one centre nationally in Ankara serving a very large geographical region).^28^ By advocating for an increase in the number of adequately trained providers in rural areas, this helped expand the service and improve service accessibility in underserved areas of Turkey (O10a). A similar study adapted a QoL questionnaire for SCI patients in Brazil using a Delphi method and trained community healthcare workers to carry out these assessments, which is an example of shared Theory of planned behaviour-informed shared decision making (M10b).^29^ Through task-shifting models, training non-specialist workers in basic assessment of ABSI was provided in a cost-effective way to augment otherwise overstretched services, thereby reducing workforce constraints (O10b).^29^

#### CMOC 11: Incentivisation

In LMICs, low salaries, limited professional growth and lack of career development opportunities result in higher burnout and greater healthcare worker emigration than in HICs, particularly in SCI, epilepsy and tertiary disability management (C11).^67^ Several studies have implemented workforce training programmes, yet continue to struggle with long-term retention.^28 29^ Some studies used CEI-informed workforce incentivisation strategies (certification and public recognition) for SCI specialists, which in turn fostered a greater commitment to their roles (social exchange theory (SET)) (M11a).^28^ Career ladders were also introduced to offer a clear growth pathway and structured professional advancement (M11b). This supported a sense of competence (SDT) and long-term commitment.^29^ In one Chilean study, epilepsy healthcare workers received financial incentives in a performance-based payment system, creating a reciprocity effect (SET) where workers provided better patient care as they were compensated fairly.^30^ This ultimately improved workforce volume and retention, and improved quality of long-term care (O11).

### Improving infrastructure and timely access to care

#### CMOC 12: Aligning policy with local need and expanding rural healthcare reach

LMICs often lack community representation in decision-making processes as policy design often focuses on the clinical needs of the urban communities, without embedding CEI in the policy-making process, resulting in a disconnect between policymakers and patients’ lived experiences (C12).^68 69^ More specifically, adaptations required to overcome centralisation of services, transportation barriers (such as terrain, climate and distance) for patients with ABSI in rural areas and LMICs are often not adequately considered. CoP-driven multistakeholder national taskforces including policymakers, government legislative representatives, relevant NGOs and patients have been implemented in various LMIC settings.^28^,^30^ For example, one study implemented satellite-supported mobile clinics which provided epilepsy care for rural communities. By involving local champions, early adoption and uptake of rural health services was accelerated achieving widespread acceptance through DIT-informed knowledge exchange (M12).^62^ Through these nationwide efforts, more equitable, community-informed policies were generated (O12).

### Descriptive patterns in CEI implementation across studies

In addition to the CMOC synthesis above, common patterns in how CEI strategies were implemented into practice emerged. These patterns are detailed below to highlight the approaches taken to the design and implementation, and governance of CEI in ABSI research.

### Design and implementation

Alavinia *et al* recognised that return-to-work (RTW) following SCI is influenced by 11 key factors including vocational counselling, social support, and age.^41^ People living with SCI lack dedicated vocational rehabilitation, which is key to RTW. The SCI-High project and interdisciplinary ‘working group’ aimed to address this using a modified Delphi process to build consensus on key facilitators and barriers to employment.^42^ This resulted in three-tiers of indicators: structure (presence of employment centres), process (employment assessments) and outcome (RTW scale) which can be used to measure employability across all Canadian rehabilitation programmes.^42^ Similarly, Benn *et al* implemented CEI longitudinally, using Spinuzzi’s model of involvement in exploration, discovery and prototyping stages to design a visual feedback balance tool.^38^

An RCT by Douglas *et al* included CEI where a CAB was used to co-develop the robotic-gait exoskeleton for SCI rehabilitation, refine it iteratively at each phase of the study, then review its outcomes.^36^ Multiple changes were implemented as a direct result of the CAB including assessing affective outcomes and tailoring the recommended 90 min of robotic gait training by patient tolerance. One study found that a multicentre SCI knowledge mobilisation network (SCI KMN) built implementation science capacity but did not evaluate the sustainability of best practice implementation and therefore was vulnerable to withdrawal of government support.^44^ Therefore, a more updated consortium, SCI Implementation and Evaluation Quality Care Consortium helped affiliate with international and non-government organisations identifying shared core values and subsequently strengthening the vision. In one study, long-term sustainable implementation of change was driven by a dedicated diverse stakeholder group: ‘SCI Implementation and Evaluation Quality-Care Consortium’ in Ontario.^42^

### Governance, research priorities and community collaboration

Central to CEI is the shared leadership approach advocating for IKT rather than superficial ‘tokenistic’ consultation as described by Biller *et al.*^33^ The degree of involvement and quality of CEI itself was measured using the ‘Stakeholder-centric Engagement Evaluation’ tool, and dedicated training was offered to community-members to build confidence and empower them to contribute meaningfully to research.^40^ Segovia *et al* emphasised that community stakeholders can take effective lead roles in lobbying efforts for social and infrastructural reform with system-level impact to improve the quality of life of patients with refractory epilepsy in Chile.^30^ Additionally, equitable participation of groups in CEI is important to ensure a balanced representation of clinical needs and perspectives.^36^ Wolfe *et al* adopted a five-round Delphi method involving different stakeholders well-balanced in demographic characteristics to develop pressure-ulcer guidelines for patients with SCI.^37^

### Programme theory

**Figure 2:**
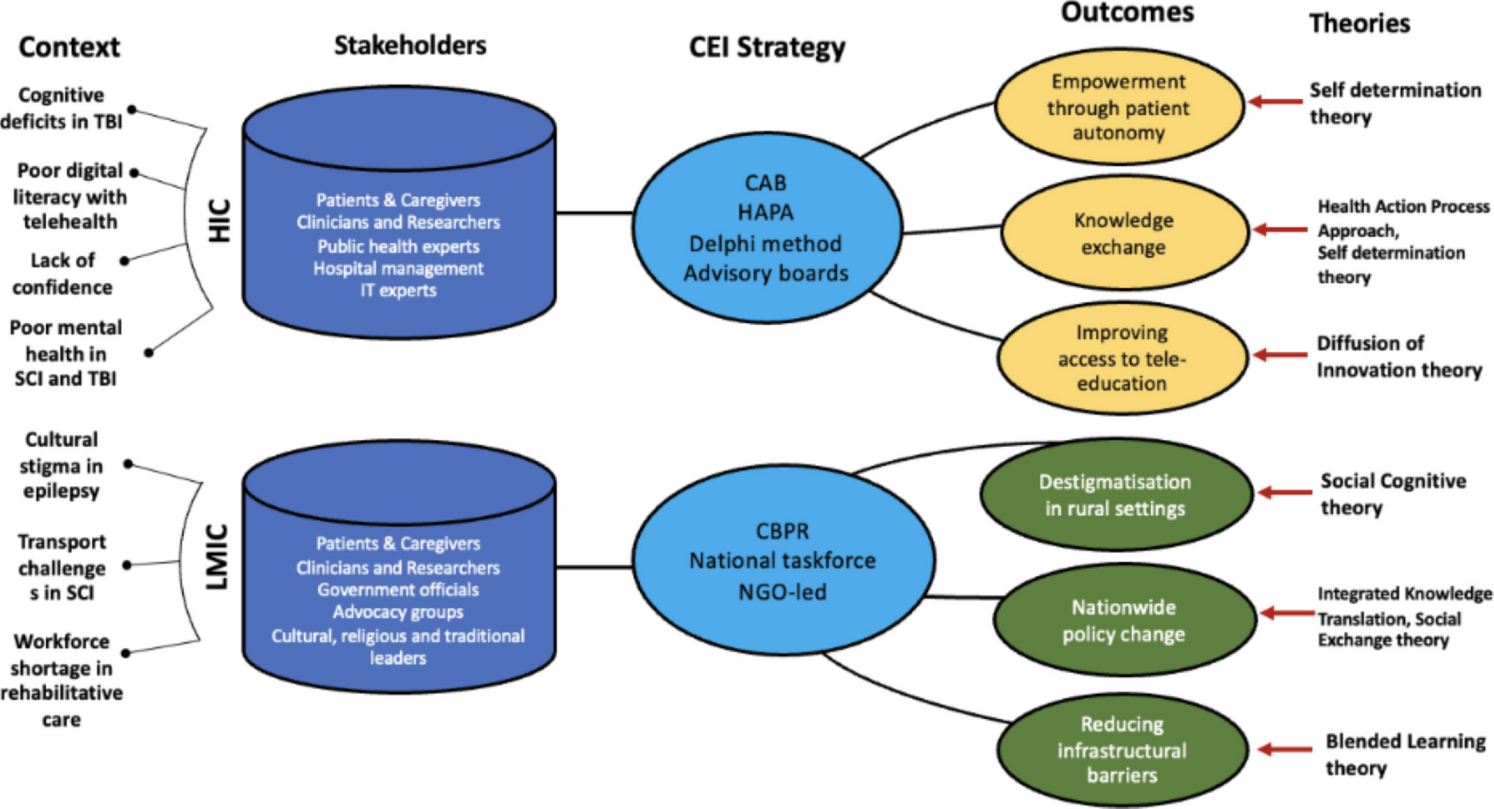
Programme theory. *CAB, community advisory board; CBPR, community-based participatory research; CEI, community engagement and involvement; HAPA, Health Action Process Approach; HIC, high-income country; IT, information technology; LMIC, low and middle-income country; NGO, non-governmental organisation; SCI, spinal cord injury; TBI, traumatic brain injury.

The programme theory synthesises the 12 key CMOCs above illustrating the key differences between HICs and LMICs (figure 2).

## Discussion

Findings from 17 studies illustrated that in HICs, healthcare systems are often well-resourced but limited by a high degree of fragmentation, leading to disempowerment, lack of understanding and poor uptake of growing telehealth platforms, particularly affecting those with cognitive deficits post-TBI.^69 70^ The higher degree of social isolation in HICs poses unique challenges to the use of peer-support or peer-facilitated reintegration for those with SCI rehabilitation needs.^32 39 54^ Through integrated CABs and HAPA goal-setting models, expert patients from SCI and TBI populations can positively influence the co-design of clinical interventions and action plans for those with ABSI.^38^ CBPR and peer-support networks use SCT to promote observational learning and leverage shared experiences which improve motivation and sustainable patient-led engagement in SCI and TBI services.^32 71 72^

Findings from the five studies showed that, in LMICs, healthcare engagement is curtailed by workforce shortages and infrastructural limitations which limit access to post-ABSI care.^26^,^30^ Community reintegration and cultural acceptance, specifically in epilepsy populations, is differentially hindered by high rates of social stigma.^73^ Engagement of respected community figures and local advocacy groups as key stakeholders in nationwide policymaking initiatives was shown to facilitate more equitable care with improved healthcare coverage in underserved areas.^27 30 74^ Additionally, through IKT-led adaptation of national interventions to address local need, more effective and culturally relevant services can be provided for patients with SCI and epilepsy.^27 39^

Following higher-level interpretation of the refined CMOCs identified, explanations from the literature that apply across several CMOCs together were postulated. These can be grouped into three overarching themes that capture the key methods CEI strategies contribute to positive outcomes across various ABSI contexts: (1) empowerment and motivation, (2) knowledge exchange and capacity building and (3) destigmatisation and social inclusion.

### Theme 1: Empowerment and motivation

Empowerment and self-determination emerged as a central mechanism in the CEI strategies of 40% of included studies, particularly driving self-efficacy and motivation in patients with ABSI.^2831 33 37^,^39 42 43 45^ The concept of empowerment is rooted in SDT emphasising autonomy and competence as foundational to motivation.^75^ Kukla *et al* concluded that when patients are treated as active agents in their rehabilitation programme through co-design, goal-setting and peer-mentoring, they are more likely to engage in sustainable health behaviours.^76^ This improves psychological well-being as well as treatment adherence and physical health.^77^,^79^ Motivational interviewing and participatory goal planning in particular are two methods reducing perceived helplessness and community reintegration in patients with TBI as the CEI methods support their sense of agency.^53^

### Theme 2: Knowledge exchange and capacity building

Another theme which reflected a hallmark of CEI effectiveness in seven studies was structured knowledge exchange between patients, researchers and community representatives.^27 31 34 36 38 40 46^ KT frameworks focus on tailoring information to the context unique to the specific target population.^80^ CoP models or stakeholder advisory boards have been shown to enhance system responsiveness in ABSI communities and reduce fragmentation of services.^81^ The bidirectional nature of co-learning processes ensures the transfer of experiential and clinical knowledge which develops the contextual and cultural grounding of the interventions.^82^

### Theme 3: Destigmatisation and social inclusion

In the five studies from LMICs and one study from an Aboriginal group in Australia, stigma and social exclusion for patients with ABSI remains a significant challenge.^26^,^3047^ CEI strategies often used SCT to challenge stigma through social-modelling and observational learning.^38 46^ Involving respected community figures and peer-role models can shift societal attitudes and reduce stigma which improves social acceptance.^83^ In disability-inclusive developments, programmes with CEI are more successful in addressing stigma as they give marginalised communities a voice to share their experience; a core component of destigmatising clinical care and policy.^84 85^

### Dearth of CEI in neurosurgical literature

There is no clear consensus in the current literature to explain the dearth of CEI in neurosurgical literature. Historically, investigator-centric studies have predominated in neurosurgical research prioritising clinical and procedural outcomes rather than CEI.^1^ Nevertheless, this could be attributable to a number of factors. The acute and time-critical nature of ABSI frequently results in patients lacking capacity and requiring urgent intervention. This can limit opportunities for patients to engage in decision-making about trial design or intervention delivery.^22^ Similarly, patients with TBI often suffer cognitive impairment limiting their capacity for sustained involvement in research delivery.^22^

### Strengths and limitations

This realist review exhibits several strengths. The methodology follows the RAMESES II guidelines employing an iterative and comprehensive search strategy, systematically searching four databases and the grey literature (online supplemental file 1). The use of CMOCs facilitated careful exploration of the complexity of the CEI interventions which enabled insights beyond traditional one-dimensional temporal relationships. Furthermore, by collaborating with realist researchers (KJ and SN) to validate the IPT, the programme theory was made more credible. This iterative approach enabled refinement and expansion of the search strategy to improve representation of studies from LMICs (by searching GIM database) yielding five further studies.^26^,^30^ Although subjectivity is present in realist syntheses, the use of CMOCs, iterative refinement of the programme theory and consultation with realist experts helped reduce potential interpretive bias. Additionally, this study was supported by clinical academics with vast expertise in ABSI research, both in the UK (AGK, PJH and CJW) and in LMICs (AT, AK and AFK), providing interpretive insights into the results and additional contextual understanding of the CMOCs. Finally, by stratifying the analysis by HIC and LMIC, this study provides novel insights into how CEI design is influenced by contextual health system factors (table 5).

**Table 5:** Summary of findings by HICs versus LMICs

Income index	Disease process	Contexts	Mechanism by theory	Outcomes
HIC	SCI/TBI	A focus on the use of technology (eg, tele-education) and piloting novel digital technologies. Leveraging diverse stakeholder input to develop sustainable rehabilitation programmes or changes to policy. A focus on strategies of behaviour-change centred around self-efficacy motivation-based approaches.	Self-determination theory Social cognitive theory Behaviour change theory Empowerment theory Experiential learning theory Goal-setting theory Health action process approach	CEI strategies: CABs, Delphi studies, CBPR and participatory co-design. Outcomes: focused on improving patient autonomy and gaining insight from advocacy organisations to tailor services.
LMIC	SCI/Epilepsy	Recognition of poor healthcare infrastructure and access to treatment requiring more high-level interventions, for example, government personnel. A focus on destigmatisation due to poor health literacy and public understanding of neurosurgical disease. Solutions are community-focused, however, more policymaker-driven to enable systemic change.	Social cognitive theory Diffusion of innovation theory Theory of planned behaviour	CEI strategies: task forces, public education campaigns, IKT, participatory co-design. Outcomes: focused on destigmatisation and finding culturally relevant solutions, improving healthcare access and recognising end-user barriers.

CAB, community advisory board; CBPR, community-based participatory research; CEI, community engagement and involvement; HICs, high-income countries; IKT, integrated knowledge translation; LMICs, low- and middle-income countries; SCI, spinal cord injury; TBI, traumatic brain injury.

Nevertheless, there were a few limitations to the study. There was heterogeneity in the terminology and definitions of CEI across studies, which may have influenced study identification and data synthesis, potentially affecting transferability. Exclusion of non-published literature introduces a potential reporting bias and underrepresentation of unpublished community-led initiatives. Despite inclusion of the GIM database and grey literature, there remains a relative paucity of peer-reviewed CEI studies from African countries, which may reflect publication bias or underreporting. This limits the generalisability of the study findings across all ABSI contexts globally. Boum *et al* attributed this paucity to structural barriers in research funding and epistemic injustice faced by LMIC researchers.^86^ As such, CEI practices may not be fully represented in the available literature, particularly at a community level.

### Implications for research and practice

The results of this realist review are relevant to researchers, clinicians and policymakers working to deliver care for people living with ABSI. First, embedding CEI in the early development of research will help align the design and interventions with the lived experiences, geographical contexts and cultural priorities of ABSI patients. This is particularly important in LMICs where stigma, restricted access and limited rehabilitation workforce remain challenges. The attrition rate and non-compliance with the proposed research may improve through relevance, acceptance and sustainability should key community stakeholders become collaborators. In research, consistent reporting of CEI strategies is paramount to evaluate how different CEI models influence implementation, engagement and outcomes. The data support the integration of CEI frameworks into global health initiatives and national rehabilitation efforts to deliver more equitable neurosurgical and rehabilitation services.^30^

### Recommendations and take-home messages

With the findings of this review considered, the following three recommendations were made:

1. All researchers should report on CEI methods and stakeholder involvement in neurosurgical trials, in line with NIHR vision for CEI in global health research and the updated 2025 Consolidated Standards of Reporting Trials (CONSORT) Checklist for randomised trials.^87 88^ In particular, those who lead neurotrauma research in African and South-Asian settings should do so to address the dearth of CEI in LMICs.
2. Patients and families should be involved in ABSI research as co-designers of neurosurgical and rehabilitative care solutions, and not only as participants.
3. Researchers should develop sustainable knowledge hubs and regional CoPs across rural settings and LMICs, which will help implement scalable interventions tailored to local sociocultural contexts.

## Conclusions

This study highlights the dearth of CEI in ABSI research, particularly in LMICs. The integration of CEI in ABSI research supports the paradigm shift from traditional investigator-driven research to a more inclusive patient-centred and equitable approach to healthcare policy prioritising collaboration, contextual relevance and patient empowerment. Future neurosurgical research should leverage CEI strategies identified in this study to co-design and implement research interventions and better meet the needs of their patients and communities.

## Supplementary material

10.1136/bmjopen-2025-112463 online supplemental file 1

10.1136/bmjopen-2025-112463 online supplemental file 2

10.1136/bmjopen-2025-112463 online supplemental file 3

10.1136/bmjopen-2025-112463 online supplemental file 4

10.1136/bmjopen-2025-112463 online supplemental file 5

10.1136/bmjopen-2025-112463 online supplemental file 6

## Data Availability

All data relevant to the study are included in the article or uploaded as supplementary information.
