# Supplementary material for: Understanding community and patient engagement and involvement (CEI) interventions in acquired brain and spinal injuries (ABSI): a realist review
Source: BMJ Open. 2026 Jul 3;16(7):e112463. doi: 10.1136/bmjopen-2025-112463 (PMC13343019; doi:10.1136/bmjopen-2025-112463)
Supplement: online supplemental file 1 [file bmjopen-16-7-s001.docx]

# Supplementary File 1: RAMESES II Reporting Checklist

| Item | Checklist Description | Reported in Document (Y/N) | Page Number(s) |
| --- | --- | --- | --- |
| 1 | In the title, identify the document as a realist evaluation | Y | 1 |
| 2 | Abstract/summary should include: policy/programme evaluated, setting, purpose, evaluation questions/objectives, strategy, data collection and analysis methods, key findings and conclusions; may include respondents and sampling details. | Y | 3, 4 |
| 3 | Rationale for evaluation – explain purpose and implications for focus and design. | Y | 7, 8 |
| 4 | Programme theory – describe the initial programme theory underpinning the programme/policy/initiative. | Y | 8, 9 |
| 5 | Evaluation questions, objectives and focus – state evaluation questions and objectives and how programme theory defined scope. | Y | 8, 9 |
| 6 | Ethical approval – state whether ethical approval was required/obtained and provide details or justification if not required. | Y | 2 |
| 7 | Rationale for using realist evaluation – explain why this approach was chosen/adapted. | Y | 7, 8 |
| 8 | Environment surrounding the evaluation – describe the context in which the evaluation took place. | Y | 6-8 |
| 9 | Describe the programme, policy, initiative or product evaluated. | Y | 6-8 |
| 10 | Describe and justify the evaluation design (what was planned, done and why). Provide link/reference if design not included. | Y | 8-12 |
| 11 | Data collection methods – describe and justify methods and how they contributed to programme theory development or refinement. | Y | 8-12 |
| 12 | Recruitment process and sampling strategy – describe recruitment and how the sample informed programme theory. | Y | 11, 12 |
| 13 | Data analysis – explain how data were analysed and how programme theory was developed, tested or refined. | Y | 11, 12 |
| 14 | Details of participants – who participated and how their data informed programme theory. | Y | 9-12 |
| 15 | Main findings – link findings to contexts, mechanisms and outcomes and show how programme theory was refined. | Y | 13-31 |
| 16 | Summary of findings – summarise key findings in relation to evaluation questions, purpose and programme theory. | Y | 32, 33 |
| 17 | Strengths, limitations and future directions. | Y | 35, 36 |
| 18 | Comparison with existing literature on similar programmes or initiatives. | Y | 33-35 |
| 19 | Conclusion and recommendations consistent with realist approach. | Y | 36-39 |
| 20 | Funding and conflict of interest – state funding source, role of funder and conflicts of interest. | Y | 40 |

Source: Wong, G., Westhorp, G., Manzano, A., Greenhalgh, J., Jagosh, J., & Greenhalgh, T.

(2016). RAMESES II reporting standards for realist evaluations. BMC medicine, 14(1), 96.

https://doi.org/10.1186/s12916-016-0643-1
