# Supplementary material for: Understanding community and patient engagement and involvement (CEI) interventions in acquired brain and spinal injuries (ABSI): a realist review
Source: BMJ Open. 2026 Jul 3;16(7):e112463. doi: 10.1136/bmjopen-2025-112463 (PMC13343019; doi:10.1136/bmjopen-2025-112463)
Supplement: online supplemental file 2 [file bmjopen-16-7-s002.docx]

**Supplementary File 2: Search Strategies**

**EMBASE**

1974 to Week 3 March 2025

(exp Neurosurgery/ OR exp brain injury/ OR exp traumatic brain injury/ OR exp spinal cord injury/ OR craniocerebral trauma.mp OR exp brain tumor/ or brain neoplasm.mp OR exp glioblastoma/ OR exp glioma/ OR exp refractory mesial temporal lobe epilepsy/ OR exp refractory epilepsy/ OR exp refractory focal epilepsy/ OR exp epilepsy/) AND (exp Community participation/ OR exp participatory research/ OR community engagement.mp)

Total: 359

**MEDLINE**

1974 to Week 3 March 2025

(exp Neurosurgery/ OR exp Brain Injuries/ OR exp Brain Injuries, Traumatic/ OR exp Spinal Cord Injuries/ OR exp Craniocerebral trauma/ OR exp Brain Neoplasms/ OR exp Glioma/ OR exp Glioblastoma/ OR exp Epilepsy/ OR exp Drug Resistant Epilepsy/) AND (exp Community Participation/ OR exp Community-Based Participatory Research/ OR community engagement.mp)

Total: 406

**APA PsycInfo**

1974 to Week 3 March 2025

(exp Neurosurgery/ OR exp Brain Injuries/ OR exp Brain Injuries, Traumatic/ OR exp Spinal Cord Injuries/ OR exp Craniocerebral trauma/ OR exp Brain Neoplasms/ OR exp Glioma/ OR exp Glioblastoma/ OR exp Epilepsy/ OR exp Drug Resistant Epilepsy/) AND (exp Community Participation/ OR exp Community-Based Participatory Research/ OR community engagement.mp)

Total: 6

**Global Index Medicus**

Inception to Week 4 March 2025

((Neurosurgery) OR (Brain Injuries) OR (Traumatic Brain Injury) OR (Spinal Cord Injury) OR (Craniocerebral Trauma) OR (Epilepsy) OR (Hydrocephalus)) AND ((Community Participation) OR (Community-Based Participatory Research) OR (community engagement) OR (Delphi Method) OR (Consensus meeting) OR (Community of practice) OR (Advisory meetings))

Total: 130.

**Grey Literature**

Inception to Week 4 April 2025

1. Google Scholar
2. Global health organisational websites

- World Health Organization (WHO)
- WHO Global Rehabilitation Alliance
- United Nations Children’s Fund (UNICEF)

1. Non-governmental organisations

- AO Spine
- International Brain Injury Association (IBIA)

Search terms used in the website search bar included combinations of:

‘Community engagement’ ‘community participation’ ‘patient involvement’ ‘participatory research’ ‘community advisory board’ ‘co-design’. In combination with ‘spinal cord injury’ ‘brain injury’ ‘traumatic brain injury’ ‘epilepsy’ ‘hydrocephalus’ ‘neurorehabilitation’ and ‘neurosurgery’

Total: 0
