# Supplementary material for: Understanding community and patient engagement and involvement (CEI) interventions in acquired brain and spinal injuries (ABSI): a realist review
Source: BMJ Open. 2026 Jul 3;16(7):e112463. doi: 10.1136/bmjopen-2025-112463 (PMC13343019; doi:10.1136/bmjopen-2025-112463)
Supplement: online supplemental file 3 [file bmjopen-16-7-s003.docx]

**Supplementary File 3: Steps of UNICEF’s community engagement model ^[51]^**

| Level of Community Engagement | Description | Degree of External Support |
| --- | --- | --- |
| 1. Inform and Mobilise | Communities are informed and mobilised to participate in addressing immediate or short-term concerns. Activities are largely driven by external organisations. | Strong external support |
| 2. Consult and Involve | Communities are consulted and involved in improving the delivery of services and programmes. | Some external support |
| 3. Collaborate | Communities actively collaborate with stakeholders to define priorities and contribute to programme design and decision-making processes. | With or without external support |
| 4. Empower | **Communities lead the development of systems for self-governance, establish priorities, implement interventions and build sustainable mechanisms for development within a broader support network.** | **Community-led with supportive partnerships** |

Source: Adapted from UNICEF Social and Behaviour Change Communication (SBC) Guidance on Community Engagement.^[51]^
