# Supplementary material for: Understanding community and patient engagement and involvement (CEI) interventions in acquired brain and spinal injuries (ABSI): a realist review
Source: BMJ Open. 2026 Jul 3;16(7):e112463. doi: 10.1136/bmjopen-2025-112463 (PMC13343019; doi:10.1136/bmjopen-2025-112463)
Supplement: online supplemental file 4 [file bmjopen-16-7-s004.docx]

**Supplementary Figure 1: Final PRISMA flow-chart**

**Identification of studies via databases and registers**

Records removed *before screening*:

Duplicate records removed

(n =135)

Records removed for other reasons (n =0)

Records identified from:

Ovid MEDLINE (n =406)

Embase (n =359)

APA PsycInfo (n =6)

Global Index Medicus (n =130)

*Total (N =901)*

**Identification**

Records screened (title screening)

(n =766)

Records excluded

(n =691)

**Screening**

Reports sought for retrieval

(n =75)

Reports not retrieved

(n =0)

Reports excluded:

Studies assessing community perspectives/attitudes qualitatively with no CEI in research (n =42)

Community participation as a clinical outcome (n =11)

Full-text reports assessed for eligibility

(n =75)

Studies included in review

(n =22)

Ovid Medline, EMBASE and APA PsycINFO (n=17)

Global Index Medicus (n=5)

Grey literature sources (n=0)

**Included**

*From:*  Page MJ, McKenzie JE, Bossuyt PM, Boutron I, Hoffmann TC, Mulrow CD, et al. The PRISMA 2020 statement: an updated guideline for reporting systematic reviews. BMJ 2021;372:n71. doi: 10.1136/bmj.n71
