# Supplementary material for: Understanding community and patient engagement and involvement (CEI) interventions in acquired brain and spinal injuries (ABSI): a realist review
Source: BMJ Open. 2026 Jul 3;16(7):e112463. doi: 10.1136/bmjopen-2025-112463 (PMC13343019; doi:10.1136/bmjopen-2025-112463)
Supplement: online supplemental file 5 [file bmjopen-16-7-s005.docx]

| Realist Analysis of Included Papers (N=22)  **Supplementary Table 1: Realist (CMO) Data Extraction Table** | | |
| --- | --- | --- |
| Context | **Mechanism (and associated Behavioural theories)** | **Outcome** |
| Studies from HICs | | |
| 1. *Carroll et al., 2024 (PPI in pre-clinical SCI research, Ireland)* | | |
| C1: Inclusion of people with SCI in pre-clinical settings is difficult for researchers and feels inaccessible and less worthwhile for patients. | C1: Inclusion of people with SCI in pre-clinical settings is difficult for researchers and feels inaccessible and less worthwhile for patients. | C1: Inclusion of people with SCI in pre-clinical settings is difficult for researchers and feels inaccessible and less worthwhile for patients. |
|  |  | O3: Reinforces and sustains the CAB as a tool to meet the institutional values. |
| 1. *Benn, 2023 (SCI: FES and VFBT Rehab Prototype, Canada)* | | |
| C1: Development of prototype requires holistic consideration of cost, safety, usability to enrol it in the clinical context. Co-design method pays due consideration to two neglected elements responsible for implementation success, 1) realistic awareness of functional requirements of end-users including technical and psychosocial barriers, and 2) Local operational factors including funding available.  C2: Inherent complexity of integrating advanced technology like FES into clinical practice requires a CEI approach. | C1: Development of prototype requires holistic consideration of cost, safety, usability to enrol it in the clinical context. Co-design method pays due consideration to two neglected elements responsible for implementation success, 1) realistic awareness of functional requirements of end-users including technical and psychosocial barriers, and 2) Local operational factors including funding available.  C2: Inherent complexity of integrating advanced technology like FES into clinical practice requires a CEI approach. | C1: Development of prototype requires holistic consideration of cost, safety, usability to enrol it in the clinical context. Co-design method pays due consideration to two neglected elements responsible for implementation success, 1) realistic awareness of functional requirements of end-users including technical and psychosocial barriers, and 2) Local operational factors including funding available.  C2: Inherent complexity of integrating advanced technology like FES into clinical practice requires a CEI approach. |
| 1. *Newman et al., 2023 (SCI: Tele-education/ self-help platform, USA)* | | |
| C1: Need for tele-education for SCI patients in the US. PHOENIX was designed to help individuals with SCI in the South Carolina community, who are underserved and face unique barriers in terms of rehabilitation, accessing proper health advice and managing their SCI (e.g. pressure ulcers, UTI), specifically as length of pre-discharge rehabilitation has fallen by over 70% in the last 40 years in the US. Additionally, with growing access to technology and misinformation on the web in high-income countries, PHOENIX emphasises accessibility and usability of relevant self-help online platforms. | *Self-efficacy theory, Community Advisory Board*  M1: This CAB task force leverages the Self-efficacy theory through involving SCI community members in the design of PHOENIX. Through this, patients will have greater relatability and higher engagement with attainable examples of self-management techniques.  M2: A diverse CAB is important in highly-educated communities of young patients in HICs, as the perceived relevance and personal health motivation are important components to consider in this community. A CAB approach builds on the Health Belief model, both by highlighting key health risks specific to SCI individuals and ensures patients are equipped with the requisite knowledge and awareness of how to deal with the problems. | **O1: Community Advisory Board (PHOENIX Task Force)**: This CAB ensured all components of PHOENIX: design, content creation, format, video scripts and visuals were relevant, valid and applicable.  **CAB Stakeholders included:**   - **SCI Community members and peer navigators(PNs)**: Helped with content and delivery ensuring it was tailored to SCI participants. The PNs also played a role in supporting the end-users through tele-health platforms once it was enrolled. - **Researchers and Health Professionals**: Ensuring scientific robustness in the SCI self-management system. - **Service providers and disability organisations**: shared invaluable insight into resource accessibility and current available services in the digital space, specific to the context. e.g. h**ighlighting the need for assistive technologies.** |
| 1. *Biller et al., 2023 (Promoting CE in SCI Research: Case example, USA)* | | |
| C1: Increased pressure from institutions (e.g. NASCIC and NIH) to embed lived experience in research processes.  C2: Recognition of the need to align Jefferson College of Rehabilitation Sciences (JCRS) values of inclusivity in research with that of NASCIC. | C1: Increased pressure from institutions (e.g. NASCIC and NIH) to embed lived experience in research processes.  C2: Recognition of the need to align Jefferson College of Rehabilitation Sciences (JCRS) values of inclusivity in research with that of NASCIC. | C1: Increased pressure from institutions (e.g. NASCIC and NIH) to embed lived experience in research processes.  C2: Recognition of the need to align Jefferson College of Rehabilitation Sciences (JCRS) values of inclusivity in research with that of NASCIC. |
| 1. *Walsh et al., 2022 (SCI: Enhanced medical rehabilitation, USA)* | | |
| C1: Patients with SCI have unique rehab needs requiring program adaptation. The local hospital’s goal of providing patient-centred care for SCI patients aligns with the function of the CAB in tailoring the intervention. Need for collaboration to ensure credibility. The task of adapting the rehabilitation program, termed enhanced medical rehabilitation (E-MR) requires an interdisciplinary and participatory approach to ensure validity and relevance.  C2: Need for Trust and Buy-In Across Stakeholders. E-MR requires credibility to ensure utilisation, interdisciplinary and patient trust. Alignment with institutional values for patient-centredness. The hospital in which Walsh et al. 2022 conducts the CAB for E-MR values patient-centredness which in turn gives the E-MR further sustainability and credibility. | C1: Patients with SCI have unique rehab needs requiring program adaptation. The local hospital’s goal of providing patient-centred care for SCI patients aligns with the function of the CAB in tailoring the intervention. Need for collaboration to ensure credibility. The task of adapting the rehabilitation program, termed enhanced medical rehabilitation (E-MR) requires an interdisciplinary and participatory approach to ensure validity and relevance.  C2: Need for Trust and Buy-In Across Stakeholders. E-MR requires credibility to ensure utilisation, interdisciplinary and patient trust. Alignment with institutional values for patient-centredness. The hospital in which Walsh et al. 2022 conducts the CAB for E-MR values patient-centredness which in turn gives the E-MR further sustainability and credibility. | C1: Patients with SCI have unique rehab needs requiring program adaptation. The local hospital’s goal of providing patient-centred care for SCI patients aligns with the function of the CAB in tailoring the intervention. Need for collaboration to ensure credibility. The task of adapting the rehabilitation program, termed enhanced medical rehabilitation (E-MR) requires an interdisciplinary and participatory approach to ensure validity and relevance.  C2: Need for Trust and Buy-In Across Stakeholders. E-MR requires credibility to ensure utilisation, interdisciplinary and patient trust. Alignment with institutional values for patient-centredness. The hospital in which Walsh et al. 2022 conducts the CAB for E-MR values patient-centredness which in turn gives the E-MR further sustainability and credibility. |
| 1. *George et al., 2022 (Care Free Me TBI/SCI Neuro-Rehab, Australia)* | | |
| C1: No program exists in Australia to support TBI and SCI patients with driving cessation and their mobility afterwards. There is a lack of awareness and confidence with the available modes of transport in Adelaide for these patients.  C2: Need for a tailored community mobility program as part of existing rehab services. No personalised mobility programs in existing community rehab services. Existing rehabilitation services in urban areas of Adelaide lack tailored community component specific to the needs and real-world challenges faced by their community. | C1: No program exists in Australia to support TBI and SCI patients with driving cessation and their mobility afterwards. There is a lack of awareness and confidence with the available modes of transport in Adelaide for these patients.  C2: Need for a tailored community mobility program as part of existing rehab services. No personalised mobility programs in existing community rehab services. Existing rehabilitation services in urban areas of Adelaide lack tailored community component specific to the needs and real-world challenges faced by their community. | C1: No program exists in Australia to support TBI and SCI patients with driving cessation and their mobility afterwards. There is a lack of awareness and confidence with the available modes of transport in Adelaide for these patients.  C2: Need for a tailored community mobility program as part of existing rehab services. No personalised mobility programs in existing community rehab services. Existing rehabilitation services in urban areas of Adelaide lack tailored community component specific to the needs and real-world challenges faced by their community. |
| 1. *Hitzig et al., 2021 (Indicators for community participation: SCI-High Project, Canada)* | | |
| C1: In Canada, there are disparities in access to community-based resources for SCI.  C2: No standardised, national framework for measuring or improving community participation in patients with SCI. | C1: In Canada, there are disparities in access to community-based resources for SCI.  C2: No standardised, national framework for measuring or improving community participation in patients with SCI. | C1: In Canada, there are disparities in access to community-based resources for SCI.  C2: No standardised, national framework for measuring or improving community participation in patients with SCI. |
| 1. *Alavinia et al., 2021 (Indicators of employment: SCI-High Project, Canada)* | | |
| C1: Under-supported infrastructure in Canada for re-integration of patients with SCI into the workforce.  C2: High degree of variability in quality and availability of vocational rehabilitation as a dimension of the holistic SCI rehabilitative care programs available in Canada. | C1: Under-supported infrastructure in Canada for re-integration of patients with SCI into the workforce.  C2: High degree of variability in quality and availability of vocational rehabilitation as a dimension of the holistic SCI rehabilitative care programs available in Canada. | C1: Under-supported infrastructure in Canada for re-integration of patients with SCI into the workforce.   - C2: High degree of variability in quality and availability of vocational rehabilitation as a dimension of the holistic SCI rehabilitative care programs available in Canada. |
| 1. *Bateman et al., 2021 (Experiences from SCI IEQCC, Canada)* | | |
| C1: Evidence-based rehabilitation in SCI is a growing area, however implementation is largely heterogenous due to systemic barriers e.g. lack of capacity, coordinated care etc. therefore, implementation science strategies are key for SCI care. | C1: Evidence-based rehabilitation in SCI is a growing area, however implementation is largely heterogenous due to systemic barriers e.g. lack of capacity, coordinated care etc. therefore, implementation science strategies are key for SCI care. | C1: Evidence-based rehabilitation in SCI is a growing area, however implementation is largely heterogenous due to systemic barriers e.g. lack of capacity, coordinated care etc. therefore, implementation science strategies are key for SCI care. |
| 1. *Douglas et al., 2021 (Robotic Gait Training for SCI Rehab, USA)* | | |
| C1: No standardised guidelines exist for the implementation of robotic gait training (RBT) for SCI patients requiring inpatient rehabilitation.  C2: Individuals with SCI face unique challenges during their recovery due to multiple aspects of their health, requiring a patient-centred approach. | C1: No standardised guidelines exist for the implementation of robotic gait training (RBT) for SCI patients requiring inpatient rehabilitation.  C2: Individuals with SCI face unique challenges during their recovery due to multiple aspects of their health, requiring a patient-centred approach. | C1: No standardised guidelines exist for the implementation of robotic gait training (RBT) for SCI patients requiring inpatient rehabilitation.  C2: Individuals with SCI face unique challenges during their recovery due to multiple aspects of their health, requiring a patient-centred approach. |
| 1. *Fiordelli et al., 2020 (Self-management app for SCI patients, Switzerland)* | | |
| C1: In SCI patients, pressure injuries are particularly prevalent however, patients with SCI face other barriers in self-managing these conditions.  C2: Current health apps do not involve users in their development. In particular, current clinical guidelines are poorly translated into app-based content which is applicable to daily life for community-dwelling individuals with SCI. | C1: In SCI patients, pressure injuries are particularly prevalent however, patients with SCI face other barriers in self-managing these conditions.  C2: Current health apps do not involve users in their development. In particular, current clinical guidelines are poorly translated into app-based content which is applicable to daily life for community-dwelling individuals with SCI. | C1: In SCI patients, pressure injuries are particularly prevalent however, patients with SCI face other barriers in self-managing these conditions.  C2: Current health apps do not involve users in their development. In particular, current clinical guidelines are poorly translated into app-based content which is applicable to daily life for community-dwelling individuals with SCI. |
| 1. *Wolfe et al., 2019 (SCI Needs in National policy, Canada)* | | |
| C1: Previously Canadian SCI care guidelines were limited by passive publication without embedding CEI. To establish the best practice and implement changes to national policy, a nationwide CoP was utilised. | C1: Previously Canadian SCI care guidelines were limited by passive publication without embedding CEI. To establish the best practice and implement changes to national policy, a nationwide CoP was utilised. | C1: Previously Canadian SCI care guidelines were limited by passive publication without embedding CEI. To establish the best practice and implement changes to national policy, a nationwide CoP was utilised. |
| 1. *Ma et al., 2019 (Improving physical activity in SCI, Canada)* | | |
| C1: Physical activity in SCI patients involves multiple unique barriers with a large psychological component that needs to be considered (from a patient perspective) as well as feasibile application of an intervention, translating theoretical design to real-world application (physiotherapist perspective).  C2: SCI populations have specific motivational and volitional requirements with respect to self-efficacy and are particularly affected by limited access to accessible public transport. | C1: Physical activity in SCI patients involves multiple unique barriers with a large psychological component that needs to be considered (from a patient perspective) as well as feasibile application of an intervention, translating theoretical design to real-world application (physiotherapist perspective).  C2: SCI populations have specific motivational and volitional requirements with respect to self-efficacy and are particularly affected by limited access to accessible public transport. | C1: Physical activity in SCI patients involves multiple unique barriers with a large psychological component that needs to be considered (from a patient perspective) as well as feasibile application of an intervention, translating theoretical design to real-world application (physiotherapist perspective).   - C2: SCI populations have specific motivational and volitional requirements with respect to self-efficacy and are particularly affected by limited access to accessible public transport. |
| 1. *Newman et al., 2014 (Peer-Navigator for SCI, USA)* | | |
| C1: People with SCI suffer more of preventable conditions such as pressure-injuries and UTIs. However, traditional research, including those focussed on pressure-injuries and UTIs, tends to exclude people with disabilities e.g. SCI.  C2: Specifically in South Carolina, there is a fragmented service with complexities in access to post-injury care. | C1: People with SCI suffer more of preventable conditions such as pressure-injuries and UTIs. However, traditional research, including those focussed on pressure-injuries and UTIs, tends to exclude people with disabilities e.g. SCI.  C2: Specifically in South Carolina, there is a fragmented service with complexities in access to post-injury care. | C1: People with SCI suffer more of preventable conditions such as pressure-injuries and UTIs. However, traditional research, including those focussed on pressure-injuries and UTIs, tends to exclude people with disabilities e.g. SCI.  C2: Specifically in South Carolina, there is a fragmented service with complexities in access to post-injury care. |
| 1. *Lindberg, 2013 (SCI: PPRQ Rehab Questionnaire, Sweden)* | | |
| C1: Lindberg’s objectives was to capture patient experiences and perceptions of rehab which is aligned with growing emphasis of person-centred care (PCC) principles in the validation process of clinical interventions/ questionnaires in SCI care.  C2: SCI patient needs are largely heterogenous based on injury morphology and different social contexts. Their preferences evolve both intrinsically and through improvements in care.  SCI communities in particular suffer from lack of motivation, therefore, involvement in research and sense of ownership can in turn lead to better clinical outcomes. | C1: Lindberg’s objectives was to capture patient experiences and perceptions of rehab which is aligned with growing emphasis of person-centred care (PCC) principles in the validation process of clinical interventions/ questionnaires in SCI care.  C2: SCI patient needs are largely heterogenous based on injury morphology and different social contexts. Their preferences evolve both intrinsically and through improvements in care.  SCI communities in particular suffer from lack of motivation, therefore, involvement in research and sense of ownership can in turn lead to better clinical outcomes. | C1: Lindberg’s objectives was to capture patient experiences and perceptions of rehab which is aligned with growing emphasis of person-centred care (PCC) principles in the validation process of clinical interventions/ questionnaires in SCI care.  C2: SCI patient needs are largely heterogenous based on injury morphology and different social contexts. Their preferences evolve both intrinsically and through improvements in care.   - SCI communities in particular suffer from lack of motivation, therefore, involvement in research and sense of ownership can in turn lead to better clinical outcomes. |
| 1. *Gauld et al., 2010 (Service provision for ABI Rehabilitation, Australia)* | | |
| C1: Disproportionately higher incidence of ABI in Aboriginal Australians, however, significantly under-represented in rehab services.  C2: Urban nationwide rehab programs are felt to be culturally inappropriate and inaccessible to rural Aboriginal communities. | C1: Disproportionately higher incidence of ABI in Aboriginal Australians, however, significantly under-represented in rehab services.  C2: Urban nationwide rehab programs are felt to be culturally inappropriate and inaccessible to rural Aboriginal communities. | C1: Disproportionately higher incidence of ABI in Aboriginal Australians, however, significantly under-represented in rehab services.  C2: Urban nationwide rehab programs are felt to be culturally inappropriate and inaccessible to rural Aboriginal communities. |
| 1. *De Pompei et al., 2001 (TBI Collaborative Planning Group, USA)* | | |
| C1: Due to lack of co-ordinated community care in US, patients with TBI suffer differentially of lack of follow-up. In particular, in Summit County, Ohio, some TBI patients are served by multiple agencies and some by none.  C2: There is a lack of trained personnel familiar with managing patients with TBI and inadequate case management infrastructure. | C1: Due to lack of co-ordinated community care in US, patients with TBI suffer differentially of lack of follow-up. In particular, in Summit County, Ohio, some TBI patients are served by multiple agencies and some by none.  C2: There is a lack of trained personnel familiar with managing patients with TBI and inadequate case management infrastructure. | C1: Due to lack of co-ordinated community care in US, patients with TBI suffer differentially of lack of follow-up. In particular, in Summit County, Ohio, some TBI patients are served by multiple agencies and some by none.  C2: There is a lack of trained personnel familiar with managing patients with TBI and inadequate case management infrastructure. |
| Studies from LMICs | | |
| 1. *Segovia et al., 2020 (Destigmatising Epilepsy in Chile)* | | |
| C1: Using a vessel of a large formal NGO such as LICHE to support patients and families of epilepsy in Chile, helps formalise the influence of interventions such as educational programs and public awareness which is imperative to the great challenge of destigmatising a condition and facilitating the re-inclusion of this community into society.  C2: A fragmented/ less established body such as a small single-centre consensus meeting of researchers may not mount the social influence required to change national social beliefs. | C1: Using a vessel of a large formal NGO such as LICHE to support patients and families of epilepsy in Chile, helps formalise the influence of interventions such as educational programs and public awareness which is imperative to the great challenge of destigmatising a condition and facilitating the re-inclusion of this community into society.  C2: A fragmented/ less established body such as a small single-centre consensus meeting of researchers may not mount the social influence required to change national social beliefs. | C1: Using a vessel of a large formal NGO such as LICHE to support patients and families of epilepsy in Chile, helps formalise the influence of interventions such as educational programs and public awareness which is imperative to the great challenge of destigmatising a condition and facilitating the re-inclusion of this community into society.  C2: A fragmented/ less established body such as a small single-centre consensus meeting of researchers may not mount the social influence required to change national social beliefs. |
| 1. *Tatli et al., 2019 (SCI Patient needs Questionnaire, Turkey)* | | |
| The requirement of SCI patients is comprehensive and requires a holistic approach considering physical, psychological and social reintegration barriers.  C1: Using a co-design approach aligned with self-determination theory ensures several culturally and logistically relevant adaptations to ICF core-set were made for Turkish population. This included contextualising the ICF Core set by considering patients’ viewpoints in the early post-acute rehab stage, as this is period is emphasised in the Turkish healthcare system. From those highlighted- body function e.g. bowel dysfunction/ spasticity and environmental barriers e.g. accessibility challenges were prioritised.  C2: By taking local clinicians views on-board: ICF was validated against tools being used in Turkey such as Functional Independence Measure (FIM) and Beck Depression Inventory (BDI), to allow swift integration into local clinical practice.  C3: Due to a lack of community support and rehabilitation services in Turkey for SCI patients, trained providers of ICF questionnaires were included by Tatli et al, to improve awareness and ability to assess and address SCI specific challenges. | The requirement of SCI patients is comprehensive and requires a holistic approach considering physical, psychological and social reintegration barriers.  C1: Using a co-design approach aligned with self-determination theory ensures several culturally and logistically relevant adaptations to ICF core-set were made for Turkish population. This included contextualising the ICF Core set by considering patients’ viewpoints in the early post-acute rehab stage, as this is period is emphasised in the Turkish healthcare system. From those highlighted- body function e.g. bowel dysfunction/ spasticity and environmental barriers e.g. accessibility challenges were prioritised.  C2: By taking local clinicians views on-board: ICF was validated against tools being used in Turkey such as Functional Independence Measure (FIM) and Beck Depression Inventory (BDI), to allow swift integration into local clinical practice.  C3: Due to a lack of community support and rehabilitation services in Turkey for SCI patients, trained providers of ICF questionnaires were included by Tatli et al, to improve awareness and ability to assess and address SCI specific challenges. | The requirement of SCI patients is comprehensive and requires a holistic approach considering physical, psychological and social reintegration barriers.  C1: Using a co-design approach aligned with self-determination theory ensures several culturally and logistically relevant adaptations to ICF core-set were made for Turkish population. This included contextualising the ICF Core set by considering patients’ viewpoints in the early post-acute rehab stage, as this is period is emphasised in the Turkish healthcare system. From those highlighted- body function e.g. bowel dysfunction/ spasticity and environmental barriers e.g. accessibility challenges were prioritised.  C2: By taking local clinicians views on-board: ICF was validated against tools being used in Turkey such as Functional Independence Measure (FIM) and Beck Depression Inventory (BDI), to allow swift integration into local clinical practice.  C3: Due to a lack of community support and rehabilitation services in Turkey for SCI patients, trained providers of ICF questionnaires were included by Tatli et al, to improve awareness and ability to assess and address SCI specific challenges. |
| 1. *Feniman et al., 2017 (QoL Questionnaire for SCI- QVLM, Brazil)* | | |
| C1. There is a lack of structured community care services for psychosocial SCI support, and a complementary lack of culturally appropriate tools/metrics to reliably quantify QoL in these patients (possibly bidirectionally related to the lack of educational awareness). The generic tools used are not unique to SCI or Brazilian communities.  C2. The Delphi method ensures scientific rigour, taking into account end-user concerns and incorporates an opportunity for iterative improvement. Involvement of local professionals who understand the local needs/barriers experienced by the local SCI community bridges the gaps of global instruments which may not accurately represent the local challenges, therefore providing a framework to build more patient-centred services for SCI patients in Brazil. | C1. There is a lack of structured community care services for psychosocial SCI support, and a complementary lack of culturally appropriate tools/metrics to reliably quantify QoL in these patients (possibly bidirectionally related to the lack of educational awareness). The generic tools used are not unique to SCI or Brazilian communities.  C2. The Delphi method ensures scientific rigour, taking into account end-user concerns and incorporates an opportunity for iterative improvement. Involvement of local professionals who understand the local needs/barriers experienced by the local SCI community bridges the gaps of global instruments which may not accurately represent the local challenges, therefore providing a framework to build more patient-centred services for SCI patients in Brazil. | C1. There is a lack of structured community care services for psychosocial SCI support, and a complementary lack of culturally appropriate tools/metrics to reliably quantify QoL in these patients (possibly bidirectionally related to the lack of educational awareness). The generic tools used are not unique to SCI or Brazilian communities.  C2. The Delphi method ensures scientific rigour, taking into account end-user concerns and incorporates an opportunity for iterative improvement. Involvement of local professionals who understand the local needs/barriers experienced by the local SCI community bridges the gaps of global instruments which may not accurately represent the local challenges, therefore providing a framework to build more patient-centred services for SCI patients in Brazil. |
| 1. *Tambourgi et al., 2013 (Improving legislative support for Epilepsy, Brazil)* | | |
| C1: In Brazil, epilepsy management is complex with lack of access, understanding and specific socio-economic challenges. Change requires a multifaceted approach working with academics, government representatives as well as advocacy groups to triangulate research efforts, public health aims and ground-level insights. This brings about systemic change which brings about long-lasting change to these patients in terms of confidence, stigma and social inclusion. This has been previously successful in Brazilian policy work such as National Health Care Program for Epilepsy.  Improving primary care epilepsy management by producing Caderno de Atencao Basica (Primary care handbook) | C1: In Brazil, epilepsy management is complex with lack of access, understanding and specific socio-economic challenges. Change requires a multifaceted approach working with academics, government representatives as well as advocacy groups to triangulate research efforts, public health aims and ground-level insights. This brings about systemic change which brings about long-lasting change to these patients in terms of confidence, stigma and social inclusion. This has been previously successful in Brazilian policy work such as National Health Care Program for Epilepsy.  Improving primary care epilepsy management by producing Caderno de Atencao Basica (Primary care handbook) | C1: In Brazil, epilepsy management is complex with lack of access, understanding and specific socio-economic challenges. Change requires a multifaceted approach working with academics, government representatives as well as advocacy groups to triangulate research efforts, public health aims and ground-level insights. This brings about systemic change which brings about long-lasting change to these patients in terms of confidence, stigma and social inclusion. This has been previously successful in Brazilian policy work such as National Health Care Program for Epilepsy.  Improving primary care epilepsy management by producing Caderno de Atencao Basica (Primary care handbook) |
| 1. *Min, 2003 (Improving Epilepsy Care by Taskforce, Brazil)* | | |
| C1: There is widespread stigma and insufficient community care offered for patients with epilepsy in Brazil (Campinas and Sao Jose do Rio Preto), despite its high prevalence.  Involving policy makers as well as community representatives ensures these misconceptions (including demonic possession and spiritual causes) are targeted at multiple societal levels, and importantly in this context, to ensure policymakers themselves do not hold these views.    C2: Using existing primary healthcare infrastructure ensures the interventions are wide-reaching and helps address the unpreparedness of primary care. This is particularly important as only 10-40% of epilepsy patients receive adequate medical treatment, and surgical treatment is extremely limited. The involvement of community members and training them to become community advocates is important in a context where there are no community voices/champions. | C1: There is widespread stigma and insufficient community care offered for patients with epilepsy in Brazil (Campinas and Sao Jose do Rio Preto), despite its high prevalence.  Involving policy makers as well as community representatives ensures these misconceptions (including demonic possession and spiritual causes) are targeted at multiple societal levels, and importantly in this context, to ensure policymakers themselves do not hold these views.    C2: Using existing primary healthcare infrastructure ensures the interventions are wide-reaching and helps address the unpreparedness of primary care. This is particularly important as only 10-40% of epilepsy patients receive adequate medical treatment, and surgical treatment is extremely limited. The involvement of community members and training them to become community advocates is important in a context where there are no community voices/champions. | C1: There is widespread stigma and insufficient community care offered for patients with epilepsy in Brazil (Campinas and Sao Jose do Rio Preto), despite its high prevalence.  Involving policy makers as well as community representatives ensures these misconceptions (including demonic possession and spiritual causes) are targeted at multiple societal levels, and importantly in this context, to ensure policymakers themselves do not hold these views.     - C2: Using existing primary healthcare infrastructure ensures the interventions are wide-reaching and helps address the unpreparedness of primary care. This is particularly important as only 10-40% of epilepsy patients receive adequate medical treatment, and surgical treatment is extremely limited. The involvement of community members and training them to become community advocates is important in a context where there are no community voices/champions. |
