# Supplementary material for: Understanding community and patient engagement and involvement (CEI) interventions in acquired brain and spinal injuries (ABSI): a realist review
Source: BMJ Open. 2026 Jul 3;16(7):e112463. doi: 10.1136/bmjopen-2025-112463 (PMC13343019; doi:10.1136/bmjopen-2025-112463)
Supplement: online supplemental file 6 [file bmjopen-16-7-s006.docx]

### ****Supplementary Table 2: ‘If, Then, Because’ Statements for CEI Strategies****

| Setting | Context (IF) | CEI Strategy (THEN) | Mechanism (BECAUSE) |
| --- | --- | --- | --- |
| **HICs** | 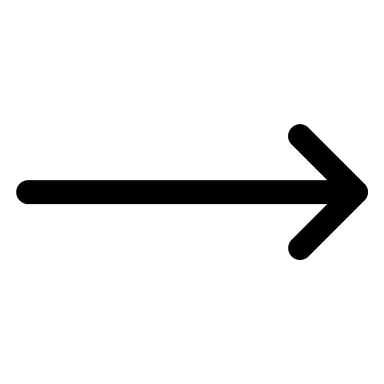Individuals with SCI experience challenges engaging with advanced technology-driven rehabilitation services | Involving patients through community advisory boards (CABs) in the co-design of tele-education platforms | 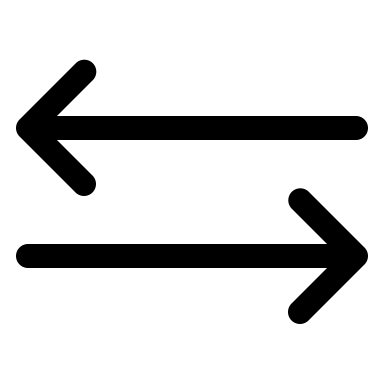Participation enhances autonomy and reduces perceived barriers to technology adoption (Health Belief Model) |
| **HICs** | 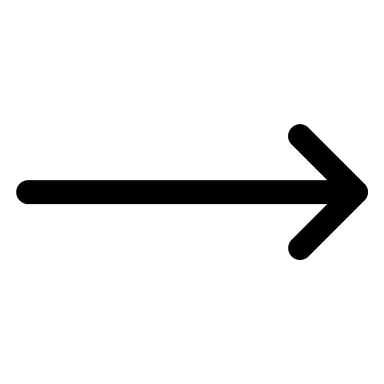Rehabilitation centres aim to improve SCI rehabilitation programmes but lack insight into patient priorities | Integrating diverse stakeholders through participatory research approaches such as consensus meetings and iterative feedback loops | 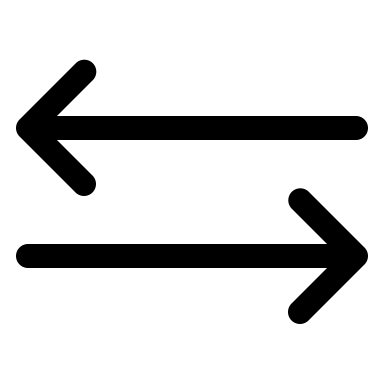Collaboration enables knowledge exchange and shared learning between clinicians, patients and researchers (Social Cognitive Theory) |
| **HICs** | 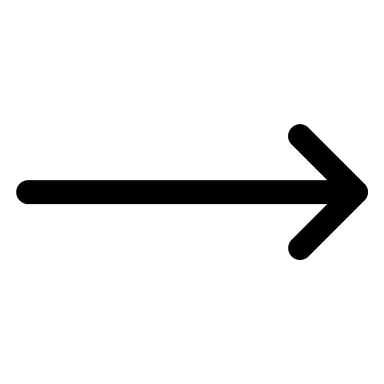Communities of ABSI patients experience low self-efficacy when engaging with rehabilitation or community services | Applying behavioural frameworks such as the Health Action Process Approach (HAPA) to guide intervention design | 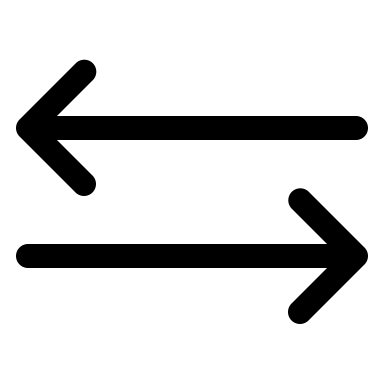Structured motivational and self-regulatory processes help translate intentions into sustained behaviour change |
| **LMICs** | 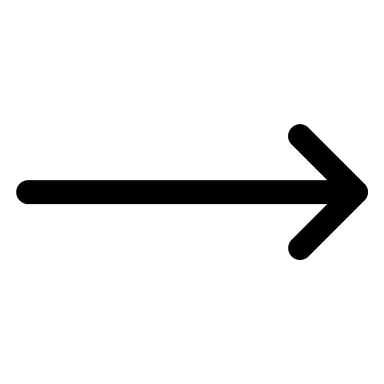Neurosurgical patients experience high levels of stigma and social marginalisation | Establishing community-based taskforces to deliver public education campaigns | 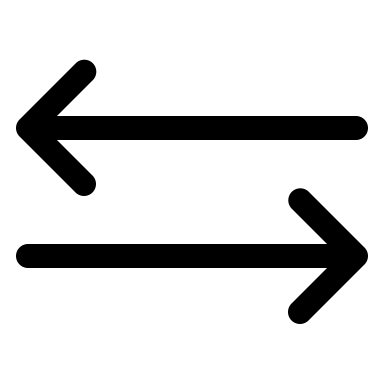Role modelling and observational learning influence social norms and reduce stigma (Social Cognitive Theory) |
| **LMICs** | 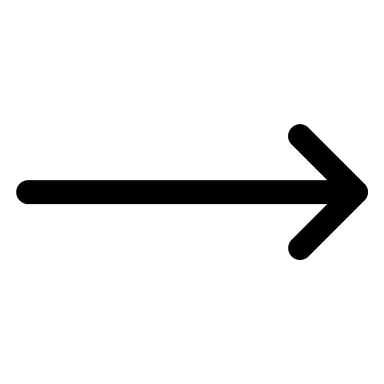Rural or LMIC communities face poor healthcare infrastructure and limited access to rehabilitation services | Using integrated knowledge translation (IKT) approaches to co-develop interventions with local stakeholders | 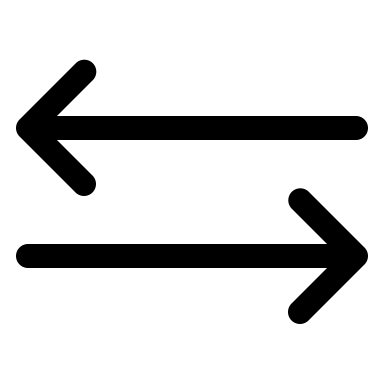Contextual knowledge from community members enables adaptation of interventions to local resource constraints |
| **LMICs** | 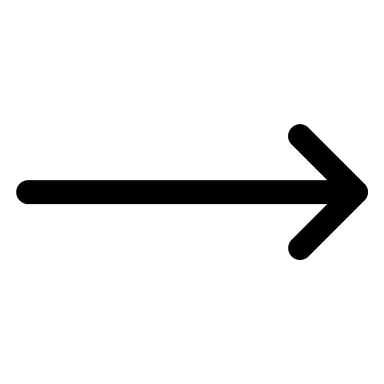Global clinical frameworks (e.g., WHO ICF Core Set) are not tailored to the local healthcare context | Applying participatory co-design approaches to adapt global tools | 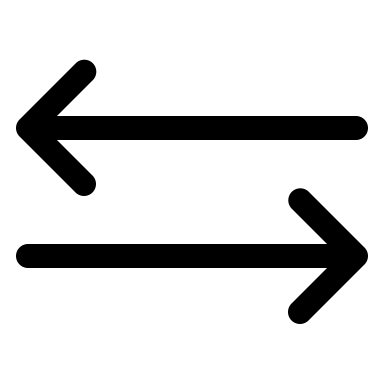Involving end-users promotes autonomy and ensures interventions reflect context-specific functioning and disability factors (Self-Determination Theory) |
