# Supplementary material for: Understanding community and patient engagement and involvement (CEI) interventions in acquired brain and spinal injuries (ABSI): a realist review
Source: BMJ Open. 2026 Jul 3;16(7):e112463. doi: 10.1136/bmjopen-2025-112463 (PMC13343019; doi:10.1136/bmjopen-2025-112463)
Supplement: Supplementary file 7 [file bmjopen-2025-112463.reviewer_comments.pdf]

## PEER REVIEW HISTORY

BMJ Open publishes all reviews undertaken for accepted manuscripts. Reviewers are asked to complete a checklist review form and are provided with free text boxes to elaborate on their assessment. These free text comments are reproduced below.

**ARTICLE DETAILS**

**Title (Provisional)**

Understanding Community and Patient Engagement and Involvement (CEI) Interventions in Acquired Brain and Spinal Injuries (ABSI): A Realist Review

**Authors**

Abdel-Fattah, Abdel-Rahman; Sescu, Daniel; Nagraj, Shobhana; Jack, Kathryn; Tirsit, Abenezer; Kumar, Anil; Khattak, Almas Fasih; Kolias, Angelos G; Hutchinson, Peter John; Whiffin, Charlotte Jane

---

**VERSION 1 - REVIEW**

---

| | |
|---|---|
| **Reviewer** | **1** |
| **Name** | **Rosyidi, Rohadi** |
| **Affiliation** | **Medical Faculty of Mataram University, Departement of Neurosurgery** |
| **Date** | **25-Oct-2025** |
| **COI** | **Yes** |

---

the author wanted to report on their research for Understanding Community and Patient Engagement and Involvement(CEI) Interventions in Acquired Brain and Spinal Injuries(ABSI): A Realist Review

The manuscript must match with the rules in the journal

Please correct the language according to the standard writing standards in English and according to the rules of the journal.

Explain the advantages and disadvantages of your research

This research is good, in presentation of research what do you need to add to your take home massage in research?

Of all your manuscripts need a lot of improvement to be published in this journal

---

| | |
|---|---|
| **Reviewer** | **2** |
| **Name** | **Farahbakhsh, Farzin** |

| | |
|---|---|
| **Affiliation** | **Tehran University of Medical Sciences** |
| **Date** | **08-Nov-2025** |
| **COI** | **None** |

---

Overall Assessment

Minor Revisions

This is an insightful and timely realist review that addresses a critical gap in the neurotrauma and neurosurgical literature—the fragmented evidence base for Community and Patient Engagement and Involvement (CEI) in ABSI. The use of the RAMESES guidelines and the stratification of findings into Context-Mechanism-Outcome Configurations (CMOCs) for HICs and LMICs is methodologically sound and provides high-value, nuanced insights.

The paper successfully clarifies the distinct challenges and effective CEI strategies in different economic settings, concluding that a shift from traditional investigator-driven research to an inclusive, patient-centred approach is necessary. The three key recommendations are strong and directly follow from the review's findings.

My main comments relate to enhancing clarity in certain methodological and discussion sections, particularly regarding the CMOC tables and the precise implications of some theoretical frameworks.

Major Comments

1. Contextual Factors and CMOC Detail

The core strength of a realist review is the clarity of its CMOCs. While the summary in Table 4 is excellent, the detailed CMOC tables (Table 3.1 and 3.2, and Supplementary Table 1) could be clearer by explicitly linking the Mechanism to the specific CEI Strategy used.

Action Required: In the manuscript's main text and CMOC tables, ensure that for each Mechanism (M), the specific CEI strategy (e.g., CAB, CBPR, Delphi method) that triggers that mechanism is explicitly named. For example, instead of "Implementing specific mechanisms such as motivational interviewing...", clarify: "Implementing HAPA-informed motivational interviewing through a CAB (mechanism)..." This improves the actionability of the findings. The current structure sometimes describes the mechanism without naming the strategy used to activate it.

2. Discussion of "Dearth of CEI" in Neurosurgical Trials

The conclusion highlights a dearth of CEI reported in neurosurgical trials. This is a critical finding, given the manuscript's focus. The discussion should dedicate a short paragraph to exploring why this dearth exists in neurosurgery specifically, beyond the general structural barriers in LMICs.

Action Required: Briefly discuss potential factors unique to neurosurgical trials (e.g., focus on acute/life-saving phases where long-term CEI is difficult to implement; high-acuity patient population post-surgery; historical investigator-centric culture) to strengthen the relevance of the three key recommendations.

3. Clarification on Role of GIM Database

The authors mention adding the Global Index Medicus (GIM) search due to a "dearth of LMIC literature" in the initial databases. This is commendable and improves global representation.

Action Required: Explicitly state how many of the final 22 included studies were exclusively identified through the GIM or grey literature searches (i.e., not found in Medline, EMBASE, or PsycINFO). This quantifies the value-add of this additional search step and reinforces the focus on LMICs. The current figure shows 766 articles identified, but a breakdown of where the final 22 came from would be helpful.

Minor Comments

LMIC/HIC Study Count Consistency: In the abstract, 22 studies are noted as meeting the criteria. The Results section mentions 17 HIC studies and 5 LMIC studies, totaling 22. This total is consistent. However, the section "Characteristics of included studies" initially mentions 17 studies met the criteria, followed by a later inclusion of 5 more studies to reach the final 22. For clarity, please state the total 22 studies earlier in the flow, perhaps by noting: "Seventeen studies were identified through database searches. Following manual searches of grey literature and reference lists, a further five studies were included for a total of 22 studies in the realist synthesis."

Theories in Figure 2: The "Theories" column in Figure 2 is extensive. To improve flow and direct interpretation, consider adding an arrow or line to visually connect the most relevant theory (e.g., Social Cognitive Theory or Health Action Process Approach) to the specific Outcome in the diagram. For example, draw a connection from Self Determination Theory to Empowerment through patient autonomy.

Citations in Discussion: The discussion section contains several sentences that summarize the LMIC findings (e.g., social isolation, marginalisation) but lack a direct citation, while the detailed context is cited in the CMOC tables. Please ensure the main findings discussed in Theme 3 are appropriately cited, linking back to the source studies.

Final Recommendation

The manuscript is a significant contribution to the literature on CEI in ABSI, particularly for its unique HIC/LMIC comparative structure. Addressing the three major comments will enhance the actionability and robustness of the findings. The minor points are mainly for presentation and clarity.

I look forward to reviewing the revised manuscript.

---

| | |
|---|---|
| **Reviewer** | **3** |
| **Name** | **Brasher, Penelope** |
| **Affiliation** | **VCHRI, Centre for Clinical Epidemiology & Evaluation** |
| **Date** | **10-Nov-2025** |
| **COI** | **None** |

---

Understanding Community and Patient Engagement and Involvement (CEI) Interventions in Acquired Brain and Spinal Injuries (ABSI): A Realist Review
This paper reports a realist review of community engagement and involvement (CEI) in acquired
brain and spinal injuries (ABSI). Data was abstracted from identified studies (n=22) and synthesized
into context, mechanism and outcome (CMO) configurations. Synthesis was performed separately
for studies from high income and low-middle income countries.
Note: Supplementary Figure 1 was not available for review.
The manuscript is well-written, but there are some opportunities to increase clarity and reproducibility. My comments are limited to my areas of expertise -- the search strategy, study
selection and data abstraction.
p 7, lns 15-16 : There is something wrong with the sentence, “In HICs, … tele-health technology.”
p 8, lns 3-5 : The sentence, “A traditional … (in this case CEI).” is unnecessary.
p 10, ln 3 : The date(s) of the search of the bibliographic databases should be provided.
p 10, ln 6 : more information about the search of the grey literature should be provided to increase
reproducibility. A brief summary should be included in the manuscript with details provided in the
supplementary material.
p 10, ln 7 : “The search was conducted by two independent reviewers …“. Two independent reviewers searching bibliographic databases (with the same search strategy) makes no sense as the
yields would be identical. Presumably the authors are referring to other processes, e.g., eligibility
assessment (title & abstract screening, full text screening), data abstraction, etc.. Please revise
following PRISMA reporting guidelines.
p 10, lns 8-11: “MeSH headings and keywords … ‘consensus meeting’.” This [partial] information is
not helpful and is not necessary as the exact search strategies are provided in the supplement.

Instead, the authors should identify the key concepts of the research question that led to the search
terms.
p 12, ln 15 to p 13 ln2 : Presumably some of this information is available in the PRISMA flowchart
and does not need to repeated here.
p 13, ln 5 : It would be helpful to include information about the steps of UNICEF's community engagement model in the supplementary material.
p 33, ln 17 : should be, "… searching four databases and the grey literature.".
Table 2, Supplementary Table 1 : Consider presenting the studies grouped by HIC/LMIC and in a
meaningful order (date of publication).

---


**Reviewer** **4**

**Name** **Sonja , Klingberg,**

**Affiliation** **Karolinska Institutet, Department of Learning, Informatics, Management and Ethics (LIME)**


**Date** **11-Feb-2026**

**COI** **None**

---

This is an interesting and ambitious realist review about community and patient engagement and involvement in the context of interventions addressing acquired brain and spinal injuries. I have no major concerns but I do have a few specific comments.

1. Consider using terms "systematic search" and "realist review" rather than systematic realist review. I can see that your search process has been systematic but the realist parts of realist evidence synthesis is often messier than a traditional systematic review and that is why it can do different things than a systematic review.

2. Think about whether the word "mechanism" is used consistently throughout the paper. At times it is used in the realist sense, but sometimes it seems to be used interchangeably with interventions or intervention components (for example "This study is a comprehensive review of the different contextual factors affecting ABSI populations in HICs and LMICs and the mechanisms through which CEI research has tackled them" does not sound like a mechanism in the realist sense of the word). Is there something more to be learned here by teasing out what exactly generates outcomes? Sometimes interventions can be part of the context, activating unexpected mechanisms and producing unintended outcomes. Sometimes using "IF, THEN, BECAUSE" statements can be useful in summarising CMOCs instead of listing contexts, mechanisms and outcomes verbally as it can help specify the exact mechanism that provides the "BECAUSE". I am not suggesting you get into another long process of theorising and refinement but changing the presentation can also sometimes bring clarity to our reasoning and interpretation.

3. Related to the previous comment, it is a little bit difficult for the reader to follow the verbal description of so many different programme theories. Tables 3.1 and 3.2 do help but consider using more visualisation or perhaps formatting the text section with the help of "IF, THEN, BECAUSE" statements or simply shorter (more abstracted/synthesised, less directly linked to specific interventions or studies) descriptions of CMOCs.

4. It was not completely clear to me where the A, B and C themes came from in the discussion section or what the purpose of them was. Perhaps just a brief sentence about what you will present to the reader in that part of the discussion can help clarify.

5. In your limitations, you refer to the synthesis as subjective. I am not sure this is a shortcoming of realist evidence synthesis - you provide a comprehensive trail of your reasoning and interpretation. Claims of objectivity in other designs are not necessarily very strong as most analyses involve choices and trade-offs that introduce elements of subjectivity (and these can be mitigated and acknowledged in different ways in different study designs). If you want to reflect on how you think subjectivity limits your review it would be great to provide the reader with some further details rather than almost dismissing realist reviews as inherently subjective in a negative sense.

I hope these comments can help make the review even more accessible to readers as realist work can be challenging to describe and can involve conflicts between clarity and depth/details.

---

## VERSION 1 - AUTHOR RESPONSE

| **Responses to comments made by Reviewer #1** | |
|---|---|
| **Comment** | **Response** |
| The author wanted to report on their research for Understanding Community and Patient Engagement and Involvement(CEI) Interventions in Acquired Brain and Spinal Injuries(ABSI): A Realist Review<br><br>The manuscript must match with the rules in the journal | We would like to thank the reviewer for the opportunity to match our study with the rules of *BMJ Open.*<br><br>We have carefully reviewed our manuscript and accompanying documents and have taken the following steps to achieve this:<br>1. Re-structured our **Abstract** (as per Editors' advice).<br>2. Included a Strengths and Limitations Box immediately following the abstract (about the methods, as per Editors' advice)<br>3. Included a *Patient and Public Involvement* statement in our **Method** section, as per Editors' advice)<br>4. Double-checked we are complying with Word Count Limit. We are slightly over the 5000 word count limit to thoroughly address the comments of the Reviewers.<br>5. Ensured Table format in editable Word documents (Number of Tables and Figures N=6 out of permissible 8). Also removed both Figures from body of manuscript into separate documents. |

| | |
|---|---|
| | 6. In addition, we have included a copy of the RAMESES II checklist with clear breakdown of where the relevant information can be located in our manuscript.<br>This document is included as a new **Supplementary File 1**, and referenced in-text accordingly. |
| Please correct the language according to the standard writing standards in English and according to the rules of the journal. | We have thoroughly reviewed the manuscript and edited the grammar, spacing, punctuation and terminology to improve readability in accordance with academic English and *BMJ Open* style.<br><br>This included, but was not limited to, correcting spacing around abbreviations, British spelling, repetitive phrasing and wrong hyphenations.<br>Please see **Manuscript with track changes,** for corrections throughout. |
| Explain the advantages and disadvantages of your research | Thank you for highlighting that we can improve the clarity of our advantages and disadvantages.<br>We have addressed your comment in three ways:<br>1. Improving our strengths and limitations sections in the **Discussion.**<br>2. Highlighting the novelty of our research in the **Introduction**.<br>3. Adding an *Implications for research and practice* section in the **Discussion.** |
| This research is good, in presentation of research what do you need to add to your take home massage in research? | We are grateful to the reviewer for highlighting the need for take-home messages in research.<br>Therefore, we have reviewed our three bulleted ***Recommendation and take-home messages*** and have ensured it includes the message we wish to convey specifically to the researchers of our readership. |
| Of all your manuscripts need a lot of improvement to be published in this journal | We thank the reviewer for highlighting the above 4 areas as improvement points for our study to be published in the journal. We hope that our comprehensive changes in response to each point uplift our manuscript to above the acceptable standard. |
| **Responses to comments made by Reviewer #2** | |
| **Comment** | **Response** |
| This is an insightful and timely realist review that addresses a critical gap in the neurotrauma and neurosurgical literature—the fragmented evidence base for Community and Patient Engagement and Involvement (CEI) in ABSI. The use of | We thank the reviewer for their accurate summary of our work and have carefully responded to the comments, in turn, below. |

| | |
|---|---|
| the RAMESES guidelines and the stratification of findings into Context-Mechanism-Outcome Configurations (CMOCs) for HICs and LMICs is methodologically sound and provides high-value, nuanced insights.<br>The paper successfully clarifies the distinct challenges and effective CEI strategies in different economic settings, concluding that a shift from traditional investigator-driven research to an inclusive, patient-centred approach is necessary. The three key recommendations are strong and directly follow from the review's findings.<br>My main comments relate to enhancing clarity in certain methodological and discussion sections, particularly regarding the CMOC tables and the precise implications of some theoretical frameworks.<br>. | |
| Contextual Factors and CMOC Detail<br>The core strength of a realist review is the clarity of its CMOCs. While the summary in Table 4 is excellent, the detailed CMOC tables (Table 3.1 and 3.2, and Supplementary Table 1) could be clearer by explicitly linking the Mechanism to the specific CEI Strategy used.<br>Action Required: In the manuscript's main text and CMOC tables, ensure that for each Mechanism (M), the specific CEI strategy (e.g., CAB, CBPR, Delphi method) that triggers that mechanism is explicitly named. For example, instead of "Implementing specific mechanisms such as motivational interviewing...", clarify: "Implementing HAPA-informed motivational interviewing through a CAB (mechanism)..." This improves the actionability of the findings. The current structure sometimes describes the mechanism without naming the strategy used to activate it. | We would like to thank the reviewer for this comment and for the opportunity to make our core strength (the CMOCs) more clear to the readership.<br><br>We have revised our **CMOC Tables 3.1 and 3.2** to be explicit with our CMOC links in line with your suggested wording- in particular the mechanism and specific CEI strategy (*see the track changed Table 3.1 and 3.2 document*).<br><br>We have also adapted the manuscript text to add clarity to these relationships. |
| Discussion of "Dearth of CEI" in Neurosurgical Trials<br>The conclusion highlights a dearth of CEI reported in neurosurgical trials. This is a critical finding, given the manuscript's focus. The discussion should dedicate a | Sure.<br><br>We have introduced a paragraph highlighting reasons for the dearth of CEI in neurosurgical literature specifically in the **Discussion** prior the *Strengths and Limitations* section. |

| | |
|---|---|
| short paragraph to exploring why this dearth exists in neurosurgery specifically, beyond the general structural barriers in LMICs.<br>Action Required: Briefly discuss potential factors unique to neurosurgical trials (e.g., focus on acute/life-saving phases where long-term CEI is difficult to implement; high-acuity patient population post-surgery; historical investigator-centric culture) to strengthen the relevance of the three key recommendations. | |
| Clarification on Role of GIM Database<br>The authors mention adding the Global Index Medicus (GIM) search due to a "dearth of LMIC literature" in the initial databases. This is commendable and improves global representation.<br>Action Required: Explicitly state how many of the final 22 included studies were exclusively identified through the GIM or grey literature searches (i.e., not found in Medline, EMBASE, or PsycINFO). This quantifies the value-add of this additional search step and reinforces the focus on LMICs. The current figure shows 766 articles identified, but a breakdown of where the final 22 came from would be helpful. | We have highlighted that the five LMIC studies came from the GIM database, and none from our grey literature searches.<br><br>We have included **Supplementary Figure 1** which details the output of each database and grey literature search.<br><br>We have also expanded **Supplementary File 2** to include the details of the grey literature search. |
| LMIC/HIC Study Count Consistency: In the abstract, 22 studies are noted as meeting the criteria. The Results section mentions 17 HIC studies and 5 LMIC studies, totaling 22. This total is consistent. However, the section "Characteristics of included studies" initially mentions 17 studies met the criteria, followed by a later inclusion of 5 more studies to reach the final 22. For clarity, please state the total 22 studies earlier in the flow, perhaps by noting: "Seventeen studies were identified through database searches. Following manual searches of grey literature and reference lists, a further five studies were included for a total of 22 studies in the realist synthesis." | We apologise that this may have caused confusion.<br><br>To address this, We have made changes to the *Search Results* section of the **Results**, by stating from the outset that there were a total of 22 studies, then explaining how arrived at this total subsequently. |

| | |
|---|---|
| Theories in Figure 2: The "Theories" column in Figure 2 is extensive. To improve flow and direct interpretation, consider adding an arrow or line to visually connect the most relevant theory (e.g., Social Cognitive Theory or Health Action Process Approach) to the specific Outcome in the diagram. For example, draw a connection from Self Determination Theory to Empowerment through patient autonomy. | We have taken your advice and updated **Figure 2** accordingly to help with the flow and visual interpretation. |
| Citations in Discussion: The discussion section contains several sentences that summarize the LMIC findings (e.g., social isolation, marginalisation) but lack a direct citation, while the detailed context is cited in the CMOC tables. Please ensure the main findings discussed in Theme 3 are appropriately cited, linking back to the source studies. | We have carefully reviewed this section, recognised this and corrected the citations. |
| The manuscript is a significant contribution to the literature on CEI in ABSI, particularly for its unique HIC/LMIC comparative structure. Addressing the three major comments will enhance the actionability and robustness of the findings. The minor points are mainly for presentation and clarity. | We would like to thank the reviewer for the positive comments on our study. We hope that our comprehensive changes in response to each point above achieve the adequate enhancement of both actionability and robustness of the findings. |
| **Responses to comments made by Reviewer #3** | |
| **Comment** | **Response** |
| This paper reports a realist review of community engagement and involvement (CEI) in acquired<br>brain and spinal injuries (ABSI). Data was abstracted from identified studies (n=22) and synthesized<br>into context, mechanism and outcome (CMO) configurations. Synthesis was performed separately<br>for studies from high income and low-middle income countries. | We thank the reviewer for this accurate and succinct summary of our study. |
| Note: Supplementary Figure 1 was not available for review. | We apologise for this omission in our previous submission, and have made sure this is available in this re-submission. |

| | |
|---|---|
| The manuscript is well-written, but there are some opportunities to increase clarity and<br>reproducibility. My comments are limited to my areas of expertise -- the search strategy, study<br>selection and data abstraction.<br>p 7, lns 15-16 : There is something wrong with the sentence, “In HICs, … tele-health technology.”<br>p 8, lns 3-5 : The sentence, “A traditional … (in this case CEI).” is unnecessary.<br>p 10, ln 3 : The date(s) of the search of the bibliographic databases should be provided.<br>p 10, ln 6 : more information about the search of the grey literature should be provided to increase<br>reproducibility. A brief summary should be included in the manuscript with details provided in the<br>supplementary material.<br>p 10, ln 7 : “The search was conducted by two independent reviewers …“. Two independent<br>reviewers searching bibliographic databases (with the same search strategy) makes no sense as the<br>yields would be identical. Presumably the authors are referring to other processes, e.g., eligibility<br>assessment (title & abstract screening, full text screening), data abstraction, etc.. Please revise<br>following PRISMA reporting guidelines.<br>p 10, lns 8-11: “MeSH headings and keywords … ‘consensus meeting’.” This [partial] information is<br>not helpful and is not necessary as the exact search strategies are provided in the supplement.<br>Instead, the authors should identify the key concepts of the research question that led to the search<br>terms.<br>p 12, ln 15 to p 13 ln2 : Presumably some of this information is available in the PRISMA flowchart<br>and does not need to repeated here. | We would like to thank the reviewer for the clear and specific comments.<br><br>We have addressed each of them in turn in the manuscript. Please see the **CEI Manuscript with track changes**.<br><br>With regards to the ‘grey literature’ comment, we have revised **Supplementary File 2** with our method of searching grey literature and expanded the detail in the methods to assure reproducibility.<br><br>We have included UNICEFs model of community engagement as **Supplementary File 3.** |

| | |
|---|---|
| p 13, ln 5 : It would be helpful to include information about the steps of UNICEF's community<br>engagement model in the supplementary material.<br>p 33, ln 17 : should be, "… searching four databases and the grey literature." | |
| Table 2, Supplementary Table 1 : Consider presenting the studies grouped by HIC/LMIC and in a<br>meaningful order (date of publication). | We have re-ordered **Table 2** in descending order of year of publication highlighted the studies from HICs and LMICs.<br><br>We have also updated **Supplementary Table 1** to reflect descending chronology. |
| **Responses to comments made by Reviewer #4** | |
| **Comment** | **Response** |
| This is an interesting and ambitious realist review about community and patient engagement and involvement in the context of interventions addressing acquired brain and spinal injuries. I have no major concerns but I do have a few specific comments. | We would like to thank the reviewer for the positive feedback and general position on our paper. We hope that our step-by-step responses below have addressed all outstanding concerns. |
| Consider using terms "systematic search" and "realist review" rather than systematic realist review. I can see that your search process has been systematic but the realist parts of realist evidence synthesis is often messier than a traditional systematic review and that is why it can do different things than a systematic review. | In recognition of the reviewers very valid comment, we have corrected this point. |
| Think about whether the word "mechanism" is used consistently throughout the paper. At times it is used in the realist sense, but sometimes it seems to be used interchangeably with interventions or intervention components (for example "This study is a comprehensive review of the different contextual factors affecting ABSI populations in HICs and LMICs and the mechanisms through which CEI research has tackled them" does not sound like a mechanism in the realist sense of the word). Is there something more to be learned here by teasing out what exactly generates outcomes? Sometimes | We thank the reviewer for highlighting the importance of using 'mechanism' consistently in its realist sense to refer to the underlying generative process by which the CEI strategy is triggered.<br><br>We have made how we use this term clear in the **Method** section, and have corrected the use of the term at several points throughout the manuscript.<br><br>We have included **Supplementary Table 2** should the readership find it helpful to express the realist logic in 'If-then-because' statements. |

| | |
|---|---|
| interventions can be part of the context, activating unexpected mechanisms and producing unintended outcomes. Sometimes using "IF, THEN, BECAUSE" statements can be useful in summarising CMOCs instead of listing contexts, mechanisms and outcomes verbally as it can help specify the exact mechanism that provides the "BECAUSE". I am not suggesting you get into another long process of theorising and refinement but changing the presentation can also sometimes bring clarity to our reasoning and interpretation. | |
| Related to the previous comment, it is a little bit difficult for the reader to follow the verbal description of so many different programme theories. Tables 3.1 and 3.2 do help but consider using more visualisation or perhaps formatting the text section with the help of "IF, THEN, BECAUSE" statements or simply shorter (more abstracted/synthesised, less directly linked to specific interventions or studies) descriptions of CMOCs. | We thank you for your advice to consider a more visually succinct adjunct such as the 'If then because' statements. Therefore, we have created **Supplementary Table 2** to help the readership digest this information. |
| It was not completely clear to me where the A, B and C themes came from in the discussion section or what the purpose of them was. Perhaps just a brief sentence about what you will present to the reader in that part of the discussion can help clarify. | Thank you for highlighting the need for some clarity around these sections.<br><br>Following the 12 CMOC descriptions in the results section, there are two groups of patterns which emerged in CEI implementation. This is a purely descriptive section with no discussion. We have updated the title of this section *Descriptive patterns in CEI implementation across studies* and added an opening sentence to help the readership understand.<br>The letters add little value and may be confusing, therefore they were removed.<br><br>In the **Discussion**, the three themes numbered 1 to 3 are focussed on the outcomes that the use of CEI serves in the ABSI population in HIC and LMICs. Again, an opening sentence prior to these themes in the **Discussion** is now included to signpost to the readership what the purpose of these themes serve and to add clarity. |

| | |
|---|---|
| In your limitations, you refer to the synthesis as subjective. I am not sure this is a shortcoming of realist evidence synthesis - you provide a comprehensive trail of your reasoning and interpretation. Claims of objectivity in other designs are not necessarily very strong as most analyses involve choices and trade-offs that introduce elements of subjectivity (and these can be mitigated and acknowledged in different ways in different study designs). If you want to reflect on how you think subjectivity limits your review it would be great to provide the reader with some further details rather than almost dismissing realist reviews as inherently subjective in a negative sense. | Following review of this comment with the wider team of realist researchers, we would like to agree with the reviewer and thank them for drawing our attention to this point of view. In view of our robust methodology, we feel that this should be merited as a strength rather than weakness. We have described it in our *Strengths* section accordingly. |
| I hope these comments can help make the review even more accessible to readers as realist work can be challenging to describe and can involve conflicts between clarity and depth/details. | We thank the reviewer for the comments, and the opportunity to make our synthesis more accessible to the readership. |
